# Sustainable shear behavior of clayey sand reinforced with recycled PET strips under moisture variation

**DOI:** 10.1038/s41598-025-32067-x

**Published:** 2025-12-15

**Authors:** Miloud Zerarka, Said Nouri, Assia Nouri, Abdelkader Kadri, Riyadh Bouddou, Bashar Tarawneh, Imane Haouam, Iryna Hunko

**Affiliations:** 1 Laboratory of Structures, Geotechnical and Risks, Hassiba Benbouali University of Chlef, Ash-Shalif, Algeria; 2 Department of Electrical Engineering, Institute of Technology, University Centre of Naama, Naama, 45000 Algeria; 3 Hourani Center for Applied Scientific Research (HCASR), Al-Ahliyya Amman University, Amman, Jordan; 4 Department of Electric Station and System, Vinnytsia National Technical University, 95 Khmelnytske shose, Vinnytsia, 21021 Ukraine; 5 The Institute of Renewable Energy of the National Academy of Sciences of Ukraine, Hnata Khotkevycha Street 20-a, 02094 Kyiv, Ukraine

**Keywords:** Cohesion, Durability, Fine-grained soils, Moisture content, PET reinforcement, Sand–kaolin mixture, Shear strength, Sustainable geotechnics, Environmental sciences, Solid Earth sciences, Engineering, Materials science

## Abstract

Moisture-sensitive granular soils containing fines, such as sand-clay (SC) mixtures, lose their shear strength after cycles of wetting and drying, compromising the stability of shallow foundations, embankments, and sub-base layers. Improving performance using conventional stabilisers such as cement and lime comes at a cost to the environment and the economy. This study examines recycled polyethylene terephthalate (PET) strips as a green reinforcement for a sand-kaolin mixture (65% sand, 35% kaolin; SC according to the unified soil classification system unified soil classification system (USCS)). Direct shear tests were performed conducted at normal stresses of 100, 200, and 300 kPa and moisture contents of ranging from 0 to 12%. The unreinforced samples suffered a loss of strength of up to 50% at 12% moisture content, while the PET-reinforced soils achieved a maximum shear stress that was 25–30% higher, 40% greater cohesion, and a 35% reduction in vertical deformation. The friction angle was slightly better (+ 1.3° at 0–4% moisture content) but decreased at higher water contents due to lubrication. Even at 12% moisture content, the reinforced soils had a cohesion of 19 kPa compared to 15 kPa for the unreinforced soils and a shear stress of 90 kPa compared to 145 kPa for the dry strength. These results confirm that PET strips act as traction elements, resisting softening due to moisture and offering a sustainable and cost-effective alternative to traditional stabilisers, while contributing to circular economy initiatives.

## Introduction

Soil is a porous and variable medium whose mechanical behavior is highly sensitive to moisture, particularly under unsaturated conditions^1,2^. Changes in moisture strongly affect shear strength, stiffness, settlement potential, and overall performance^3^. These responses depend on the interaction between soil particles, pore water, and air, which control effective stress and suction^4^. Moisture fluctuations from rainfall, seasonal cycles, or groundwater often led to strength degradation^5^. This problem is critical in fine-grained and composite soils, such as sand–clay mixtures, widely used in foundations, subgrades, slopes, and retaining structures^6–8^. Durability tests on clays produced from a mixture of PET waste and spent foundry sand demonstrated appreciable mechanical strength compared to natural clay, along with reduced water absorption, while retaining their ultimate strength after full water immersion. Compared to clay samples with a compressive strength of 18 MPa, the plastic waste-based samples exhibited an average compressive strength of 35.2 MPa. Moreover, the results revealed that the reinforced clay samples provided significant tensile resistance compared to conventional clay samples, owing to the ductility properties of recycled plastic. The acid effects were also effectively mitigated on the surface of the treated samples due to the hydrophobic nature of PET waste^9,10^. Furthermore, when incorporating recycled crushed glass, the durability results of samples subjected to complete immersion in acid solutions revealed an average increase of 70.15% in tensile strength and 54.85% in compressive strength compared to the strength values of conventional clay samples^11^. Although fiber reinforcement has advanced soil stabilization, the role of moisture variability in fine-grained soils remains poorly understood. Most studies emphasize coarse soils, saturated clays, or constant moisture, overlooking partially saturated sand–clay systems where suction and lubrication control shear response^12^. Research on PET reinforcement is particularly limited, with little quantification of its effects on cohesion, friction angle, and deformation under varying moisture contents. Yet, repurposing post-consumer PET waste as soil inclusions offers both engineering benefits and environmental value for addressing slope failures, road subgrade instability, and shallow foundation settlements^13^. More recently, bricks developed from scrap plastic waste and foundry sand have shown compressive strength values more than double those of fired clay bricks, coupled with enhanced durability under acidic and wetting–drying conditions, underscoring the engineering potential of recycled PET in construction^14^. The peak strength of the reinforced soil specimen increases gradually with an increase in dry unit weight, whereas the improvement of peak strength with moisture content occurs up to the optimum moisture content^15^. Experimental studies confirm the non-linear relationship between water content and shear strength. Water infiltration reduces matric suction and strength parameters, while increasing fines in calcareous sand lowers the friction angle under saturation^16,17^. Triaxial tests on silica sand with 20% fines showed that specimens compacted at OMC and sheared at RMC developed higher peak strength, whereas compaction at RMC followed by shearing at OMC reduced strength, highlighting the role of fines in suction and fabric^18^. The soil–water characteristic curve (SWCC) remains essential for predicting unsaturated shear strength, as suction–strength behavior may plateau or decline beyond peak suction^19^. Field data show that clayey soils in Nigeria experienced progressive settlement up to OMC, and then accelerated deformation, reflecting a shift from flocculated to dispersed structures^20^. Similarly, seasonal moisture fluctuations and groundwater changes have been linked to settlement and shear failure in fine-grained soils^21,22^. Current research has been aimed at recycling wastes like PET and foundry sand into masonry bricks to minimize dependence on natural clay and reduce environmental consequences^23^. The strength gain was, however, restricted to above 30% inclusion of PET, showing limitations in waste incorporation ratios. Recent studies have focused on the conversion of auxiliary wastes for the production of masonry bricks towards the conservation of natural clay. Recent studies have advanced the smart prediction of swelling pressure using artificial intelligence techniques. Optimized machine learning models such as XGBoost^24^, PCA/ISOMAP-ANN frameworks^25^, and ANN-based approaches applied to South African soils^26^ have all demonstrated high predictive accuracy (R²>0.92), reducing reliance on empirical correlations and conventional testing. These findings underline the growing role of data-driven tools in geotechnics, complementing experimental research and providing new insight into the moisture-sensitive behavior of expansive soils. Laboratory tests on Polish cohesive soils revealed that reducing the consistency index from stiff (IC ≈ 0.9) to soft (IC ≈ 0.3) decreased the friction angle by up to 4° and cohesion by 4–13 kPa, with stronger effects in high-clay soils. These findings underscore the need for reinforcement strategies. The conversion of industrial wastes such as fly ash, dolomitic waste, and silica fume into alternative construction materials has been highlighted as a practical application of circular economy principles, contributing to both sustainability and cost reduction in developing regions^27^. Studies on masonry bricks produced with blends of fly ash and recycled crushed glass have demonstrated improved compressive strength, durability, and resistance to sulphate attack, while simultaneously reducing reliance on natural clay resources and landfill disposal^28^. Conventional soil improvement methods, such as cement or lime stabilization, enhance strength but generate high economic and environmental costs, including greenhouse gas emissions and energy-intensive processes^29^. In many rural or low-income regions, these materials are often inaccessible or unaffordable, which underscores the need for sustainable alternatives. Recycled polyethylene terephthalate (PET), derived from abundant post-consumer plastics, offers a low-cost reinforcement option with high tensile resistance, durability, and stability. Beyond improving soil performance, PET reuse aligns with circular economy objectives by reducing plastic waste and promoting material recycling. The PET inclusions, abundant as waste, present a sustainable option, yet their effectiveness under variable moisture in fine-grained mixtures remains underexplored. This study addresses this gap by examining the shear response of sand–kaolin mixtures reinforced with recycled PET strips across different water contents and stress levels. This study investigates the shearing behavior of sand–kaolin mixed (65% sand, 35% kaolin; SC-type clayey sand) under various levels of moisture, both unreinforced and reinforced with 0.6% recycled PET strips from post-consumer waste. The dosage of reinforcement was selected based on expressed optimum performance at low dosages of fibers^30^, and the square 10 × 10 mm geometry to ensure equal soil–fiber contact^31,32^. Direct shear tests were performed at relative densities from 0% to 12% and for 100, 200, and 300 kPa normal stresses and with a relative density of 50%. The objectives of this study are to (i) assess the impact of PET reinforcement on shear strength at varying moisture levels, (ii) examine its effect on cohesion, friction angle, and vertical deformation, and (iii) provide comments on its future application as a green reinforcement material for geotechnical engineering. The article is structured as follows: Sect. 2 presents the materials and methods; Sect. 3 presents the results and discussion; and Sect. 4 presents the conclusions and recommendations for future studies.

## Materials and methods

### Soil

**Fig. 1:**
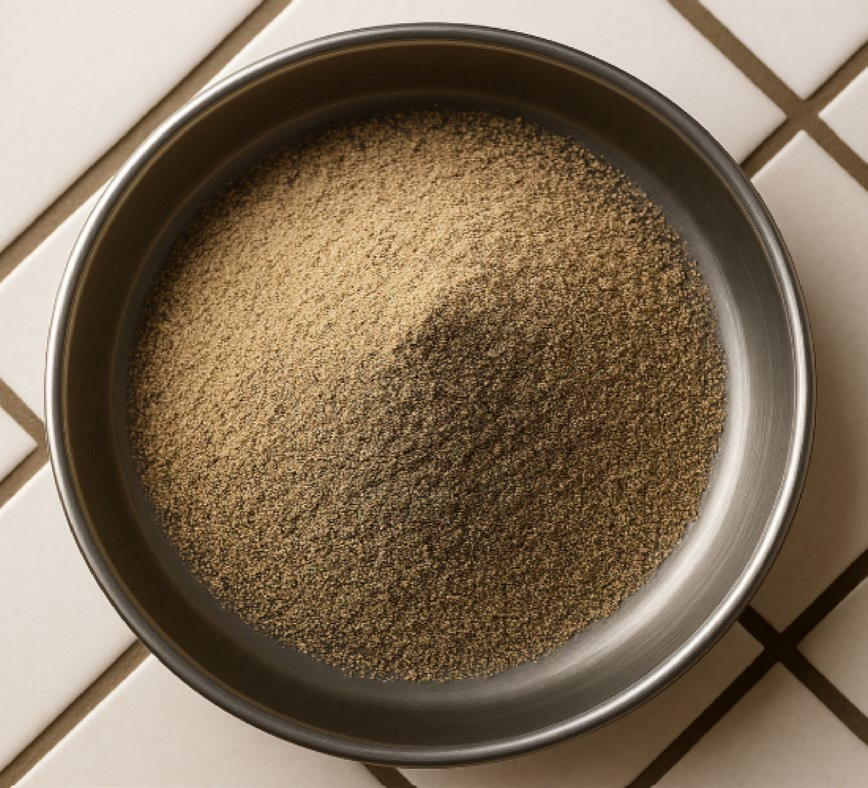
Mixture (65% sand + 35% kaolin).

**Fig. 2:**
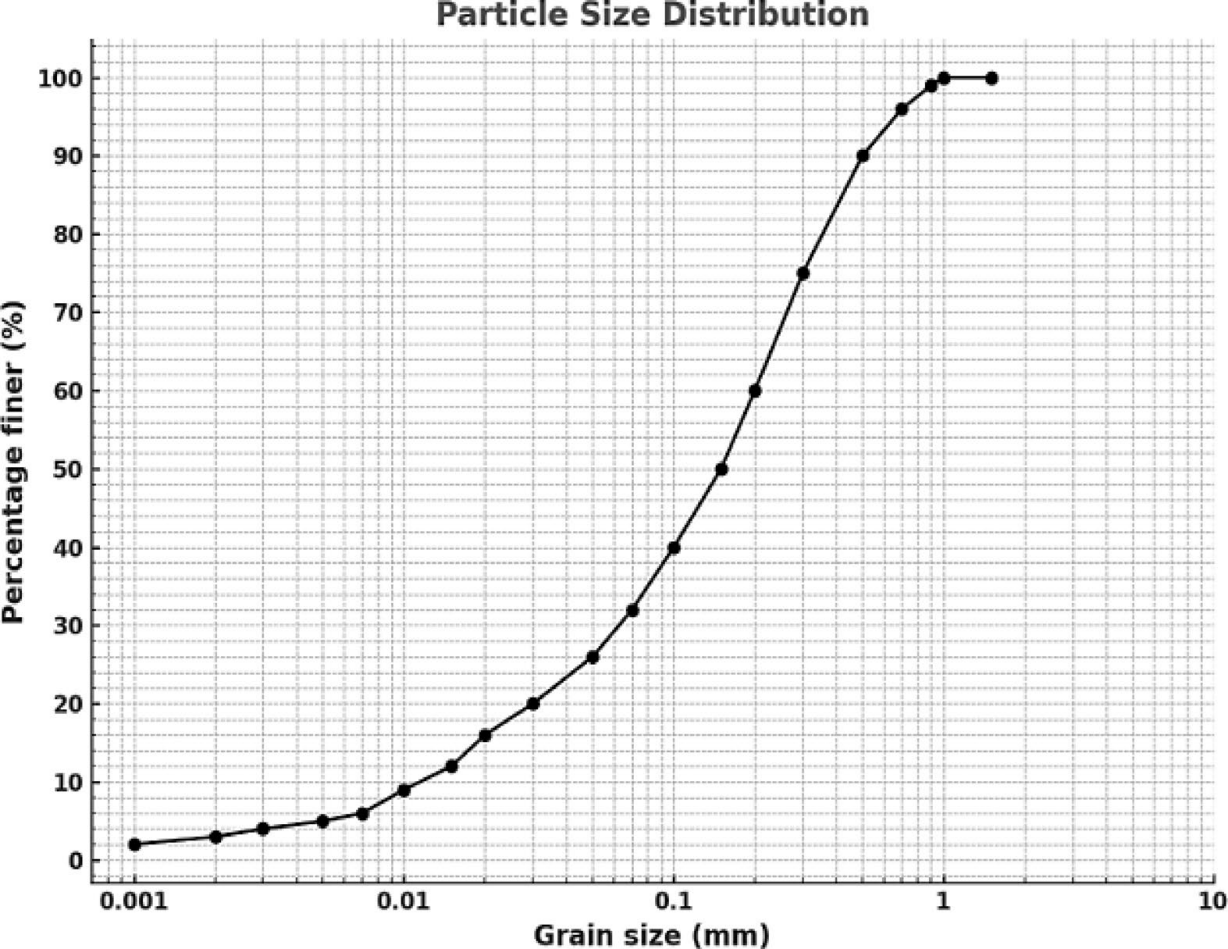
Grain size distribution curves of soil.

The soil used in this work was prepared in the laboratory by mixing 65% sand and 35% kaolin. The sand obtained from Oued Chlef in Algeria, which is characterized by its good geotechnical properties, was kaolinite used in this study, was used from Guelma. The prepared soil mixture is classified as clayey sand (SC) according to the unified soil classification system (USCS). The selected ratio of 65% sand and 35% kaolin lies within the optimal range (60–80% sand and 20–40% kaolin) commonly investigated in geotechnical studies to simulate transitional soils found in subgrades and embankments. Similar proportions were employed in previous research to assess the strength and deformation characteristics of reinforced sand–clay mixtures^33–35^. Table 1 illustrates the physical characteristics of the tested materials, Fig. 1 presents the grain size distribution curve of the soil, and Fig. 2 presents the grain size distribution curves for comparison purposes.

**Table 1:** Physical characteristics of the tested material.

	G_s_	D_10_	D_30_	D_60_	D_50_	C_u_	C_c_	LL	PL	PI
(65%sand + 35%kaolin)	2.656	0.018	0.17	0.38	0.32	21.11	4.22	20	12	8

### Plastic material

**Fig. 3:**
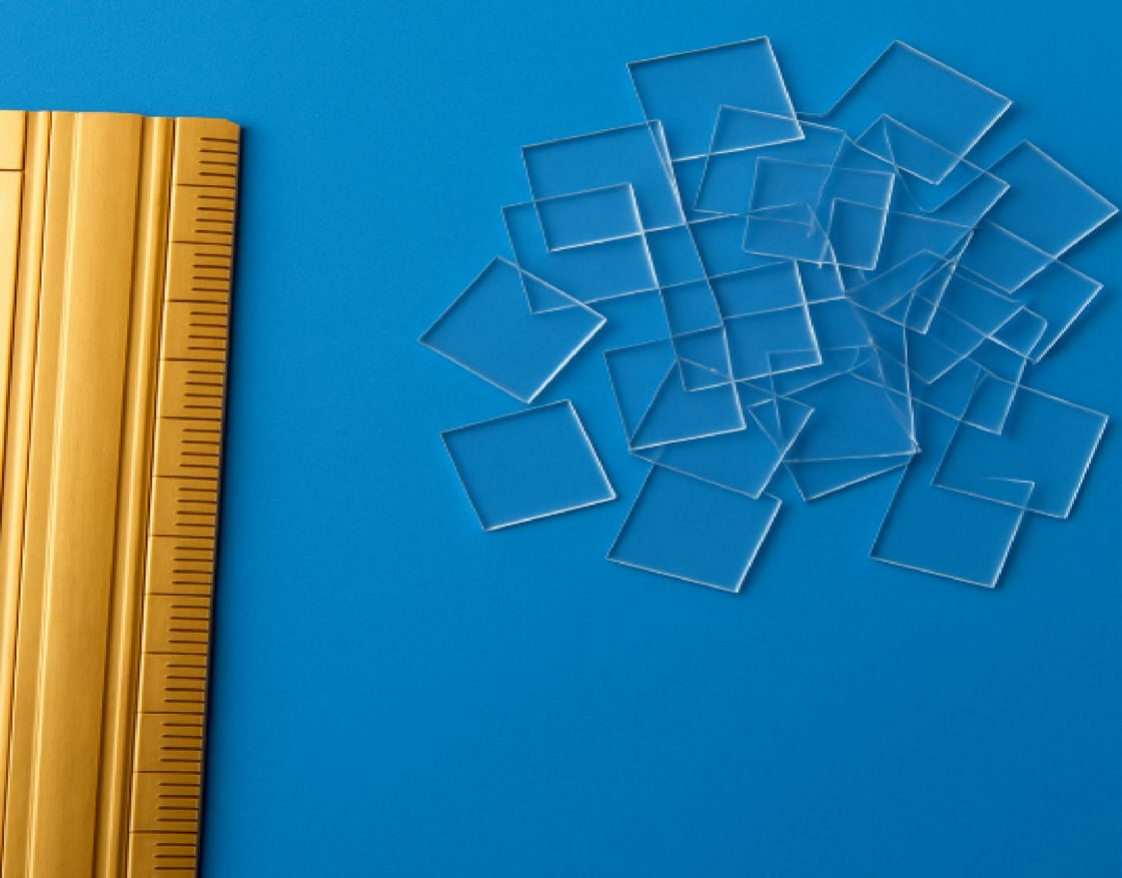
Polyethylene strips.

In this study, PET waste sourced from empty plastic water bottles collected from various cafeterias across the province of Chlef in Algeria was utilized. The PET strips have a thickness of 0.5 mm and an aspect ratio (length-to-width ratio) of one. The collected bottles underwent preparatory steps; initially, were segmented into smaller sizes and then further divided into uniform strips (10 mm*10 mm). Figure 3 presents the polyethylene strips. The selection of 10 mm x 10 mm PET strips for soil reinforcement is substantiated by their effectiveness in enhancing structural integrity while maintaining optimal soil compaction. Prior research has shown that this specific size is adequate to reinforce the soil mixture without adversely affecting compaction properties^31^. It has been demonstrated that 10 mm x 10 mm PET strips improve load distribution, thereby enhancing the soil’s shear strength through the formation of a more interconnected and stable matrix. Similarly^32^, identified this size as optimal for reinforcing clayey soil, as it effectively balances flexibility and resistance to soil displacement. Table 2 presents the physical and mechanical properties of the PET Strips used in this study.

**Table 2:** Physical and mechanical properties of PET Strips.

Property	Value
Specific gravity	1.38
Tensile strength	250–300 MPa
Elongation at break	50–120%
Melting point	250–260 °C
Young’s modulus	2.7–3.2 GPa
Strip thickness	~ 0.3 mm

### Methodology

In this study, a fine-grained soil mixture composed of 65% sand and 35% kaolin was prepared and tested under both unreinforced and reinforced conditions. The reinforcement consisted of incorporating 0.6% PET strips, by dry weight of soil, cut from recycled plastic bottles. The PET strips had a square geometry of 10 mm × 10 mm, consistent with the reinforcement geometry considered in previous studies. The selected reinforcement content of 0.6% was based on established principles of fiber-reinforced soil mechanics. In particular^30^, demonstrated that peak strength improvements in fiber-reinforced sands were most efficient at low fiber contents (0.5–0.75%), beyond which additional fibers yielded only marginal mechanical benefits while negatively affecting compaction uniformity.

#### Preparation of reinforced and unreinforced samples

The soil mixture was prepared in batches by oven-drying both sand and kaolin at 105 °C for 24 h to remove residual moisture. After cooling to room temperature, the components were thoroughly blended in the required proportions (65% sand and 35% kaolin) to ensure homogeneity. For the reinforced specimens, the PET strips were weighed to achieve 0.6% of the dry soil mass. The strips were then gradually mixed into the soil matrix using a layering and hand-turning method to promote a uniform distribution. This was followed by additional mechanical mixing to minimize clustering of strips. The effectiveness of this procedure was verified through visual inspection and random sampling of subspecimens to confirm that the PET strips were evenly distributed throughout the soil mass.

#### Compaction and relative density control

**Fig. 4:**
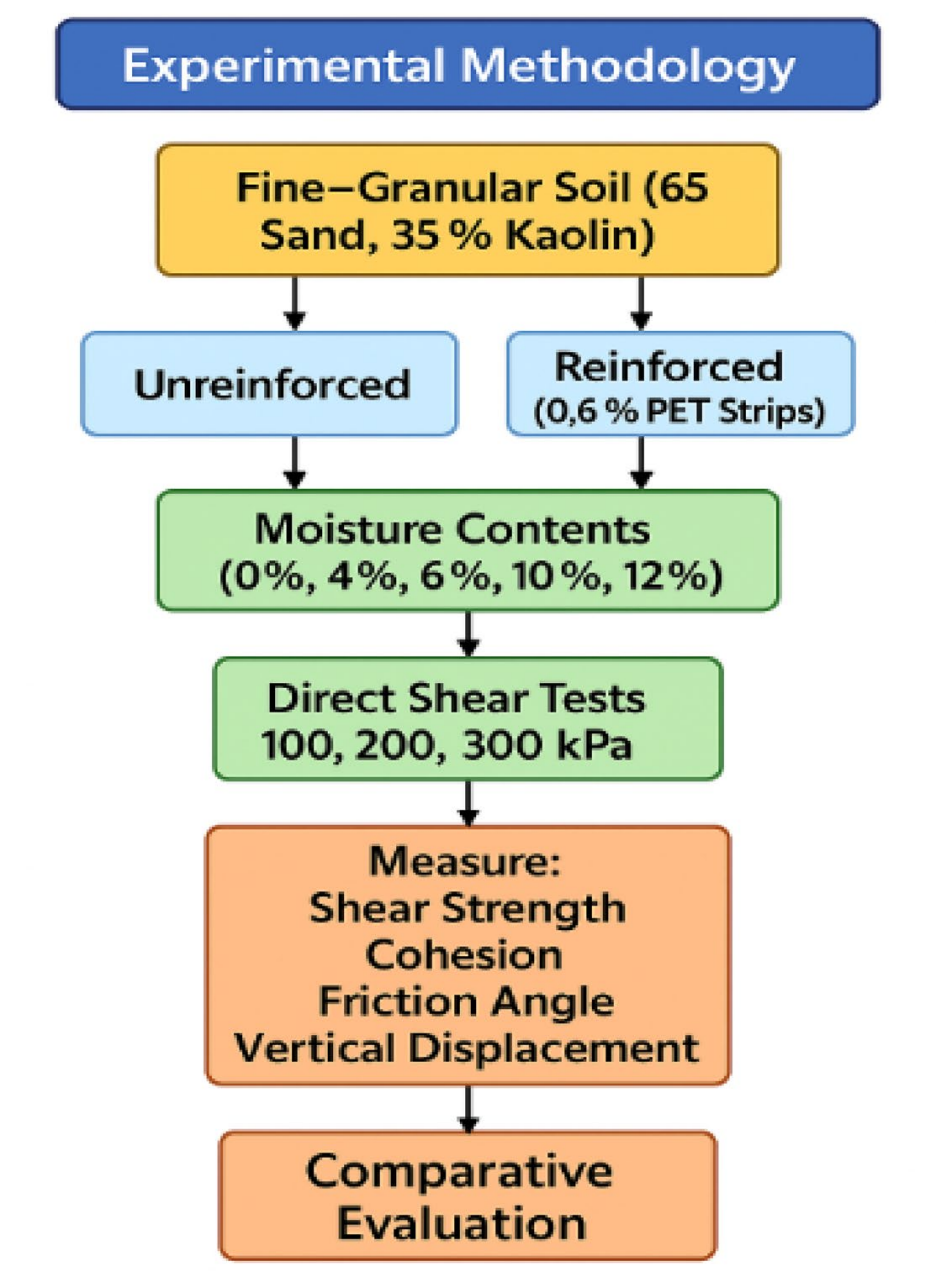
Proposal experimental procedure.

For all tests, specimens were compacted to a relative density of 50%, which corresponds to a medium-dense state. This choice was explicitly made to ensure that the soil behavior captured during shear testing would be representative and reproducible. Preliminary compaction trials indicated that relative densities higher than 70% tended to hinder the uniform distribution of PET strips, while densities below 30% resulted in excessive variability in measured strength parameters. Experimental work on geogrid-reinforced sand revealed that increasing relative density from approximately 30% to 64% significantly improved bearing capacity and reduced settlement, while low-density cases (< 30%) underperformed^36^. Likewise, studies of fiber-reinforced soils demonstrate that overly dense compaction can reduce the uniformity and orientation of reinforcement; for example, only 50–70% of fibers are aligned within ± 45° of the horizontal under very dense compaction^37^. Furthermore, broader experimental and modeling investigations support that intermediate relative densities optimize soil-fiber interaction and mechanical performance^38^. Therefore, a 50% relative density was selected as an optimal compromise—balancing reinforcement distribution and mechanical stability (Fig. 4). The target density was achieved by controlling the compaction energy applied to a known volume of soil, ensuring that the final dry unit weight corresponded to the midpoint between the maximum and minimum dry densities determined from standard compaction tests.

### Shear box dimensions and reinforcement scale consideration

**Fig. 5:**
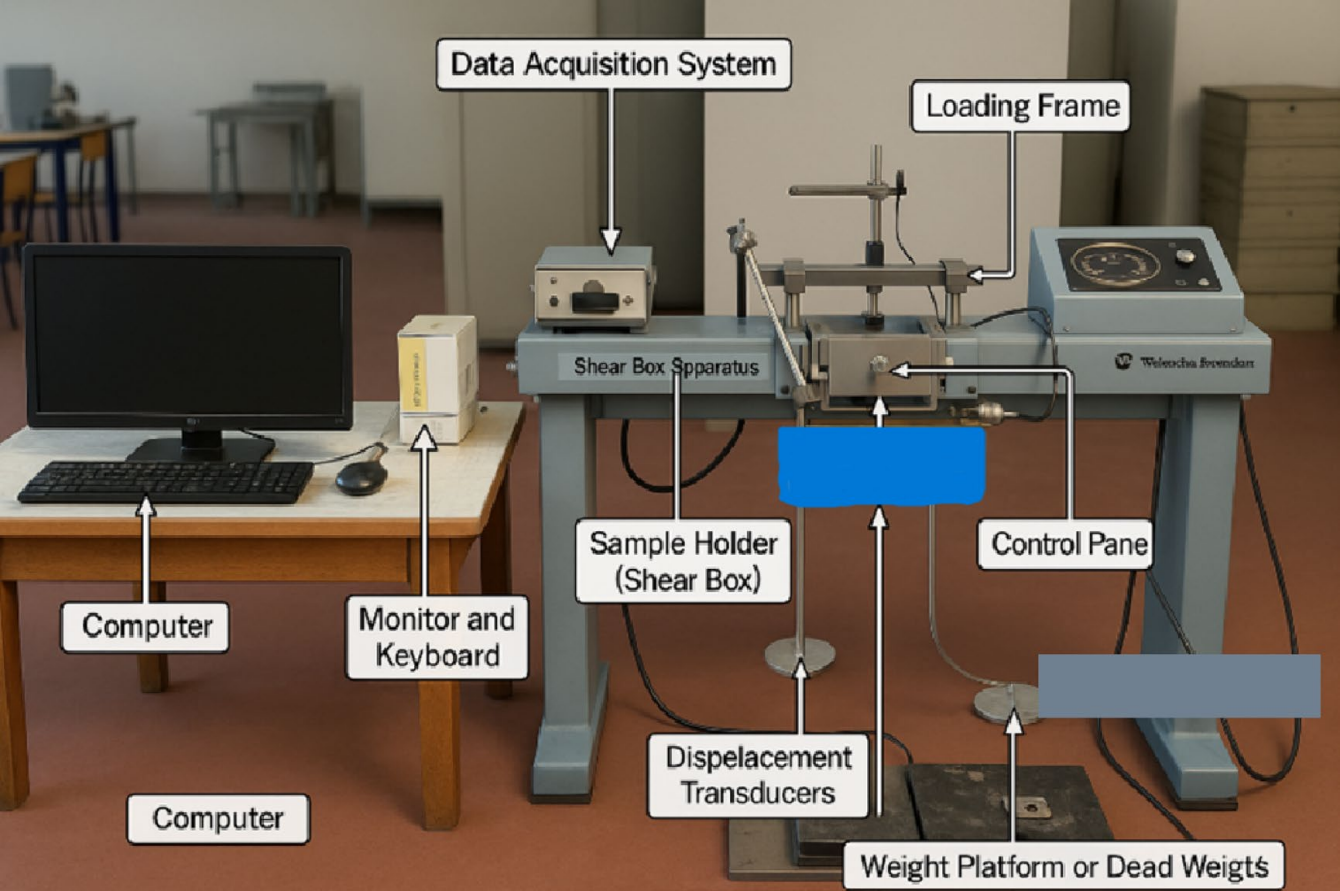
Experimental setup.

The direct shear tests were conducted using a shear box apparatus with internal dimensions of 60 mm × 60 mm × 25 mm (height). The PET strips used as reinforcement were square-shaped with dimensions of 10 mm × 10 mm, corresponding to 40% of the specimen height. This exceeds the size guideline stated in ISO 17892-10, which recommends excluding inclusions larger than 1/6 of the specimen height (approximately 4.17 mm) to minimize boundary effects. Nevertheless, in this study, the PET strips were not intended to represent coarse soil particles but rather to simulate discrete, low-percentage reinforcement elements embedded within the soil matrix, mimicking miniature geosynthetic inclusions. While this scale may introduce some non-uniformities in stress distribution, it was intentionally selected to explore the mechanical influence of PET strips in small-scale laboratory conditions. The potential implications of this size ratio are acknowledged as a methodological limitation of the experimental program (see Fig. 5). The direct shear tests were performed to identify the shear strength characteristics of an unsaturated clayey sand mixture consisting of 65% sand and 35% kaolin, reinforced with PET strips, as per the standard procedure mentioned in ASTM D 3080. The mixture proportions related to initial moisture content were set as 0%, 4%, 6%, 8%, 10%, and 12%. The mixture was allowed to settle in a sealed bag for a period of 24 h so that the initial bi-hydro mechanical conditions are attained.

The testing procedure was developed specifically for unsaturated soil, since the maintenance of matric suction is critical to ensure a correct measurement of strength values. The specimens were put inside a 60 mm x 60 mm x 25 mm shear box, consisting of three layers that were carefully compacted, applying normal stresses of 100, 200, and 300 kPa, respectively. Contrary to regular CD tests involving saturated fine-grained soils, our procedure demanded a rate designed to keep the moisture constant. Accordingly, a rate of 1.2 mm/min was adopted, as is the standard procedure involving unsaturated and reinforced soils, when preventing drainage is a key consideration^39,40^. have adopted a similar procedure when testing plastic-reinforced sand and fiber-reinforced cohesive soils, respectively. Additionally, initial tests demonstrated that this rate allowed the creation of distinct surfaces and a smooth displacement-stress plot, proving this rate suitable for maintaining unsaturated conditions during the tests.

During testing, both horizontal displacement and vertical displacement were continuously monitored, along with the applied shear load, enabling the determination of shear stress–displacement curves. Each test was repeated three times under identical conditions to confirm reproducibility, and average values were reported.

## Results and discussion

### Unreinforced samples

#### The influence of moisture content on shear strength

**Fig. 6:**
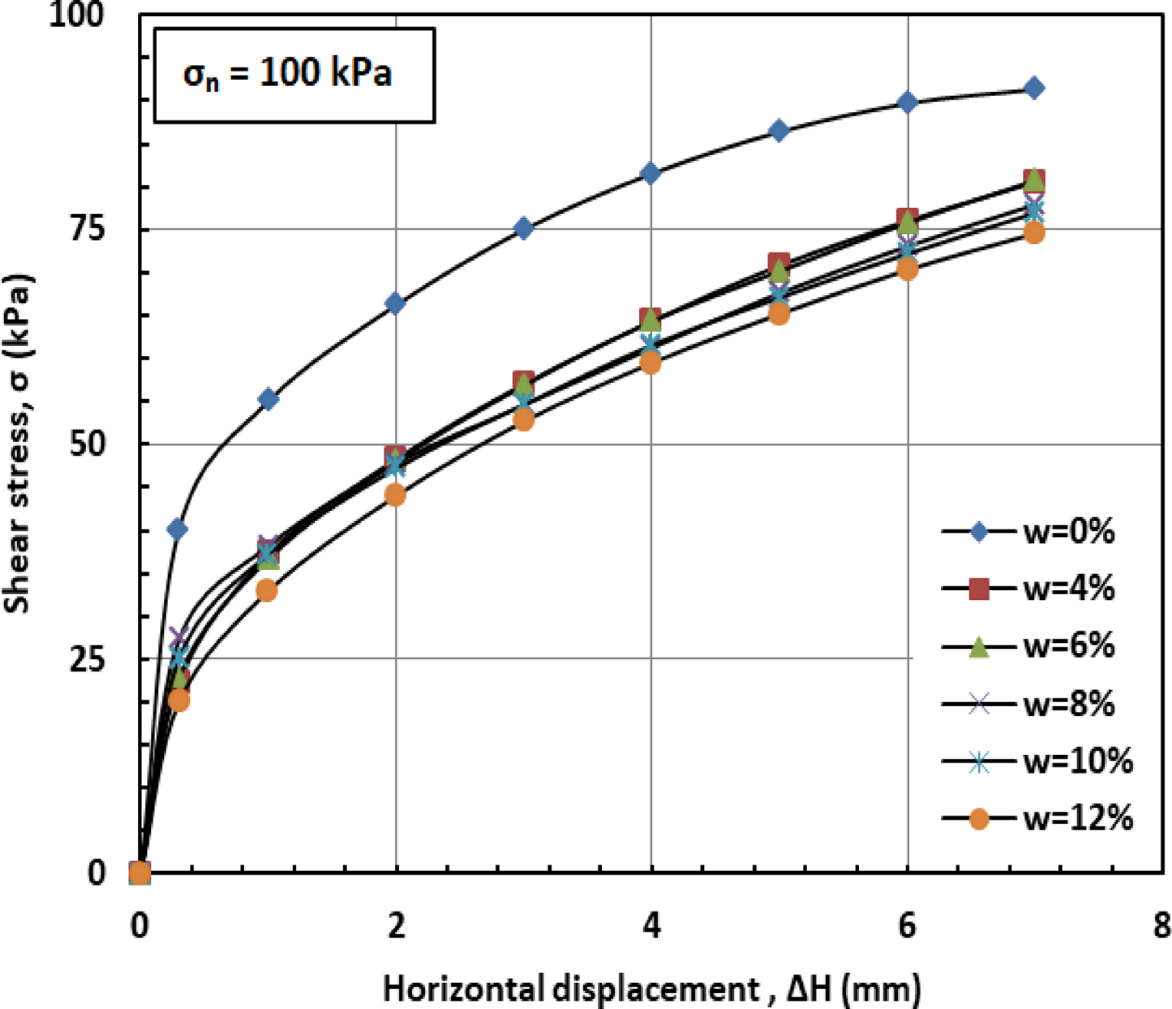
Shear strength versus horizontal displacement for 100 kPa.

**Fig. 7:**
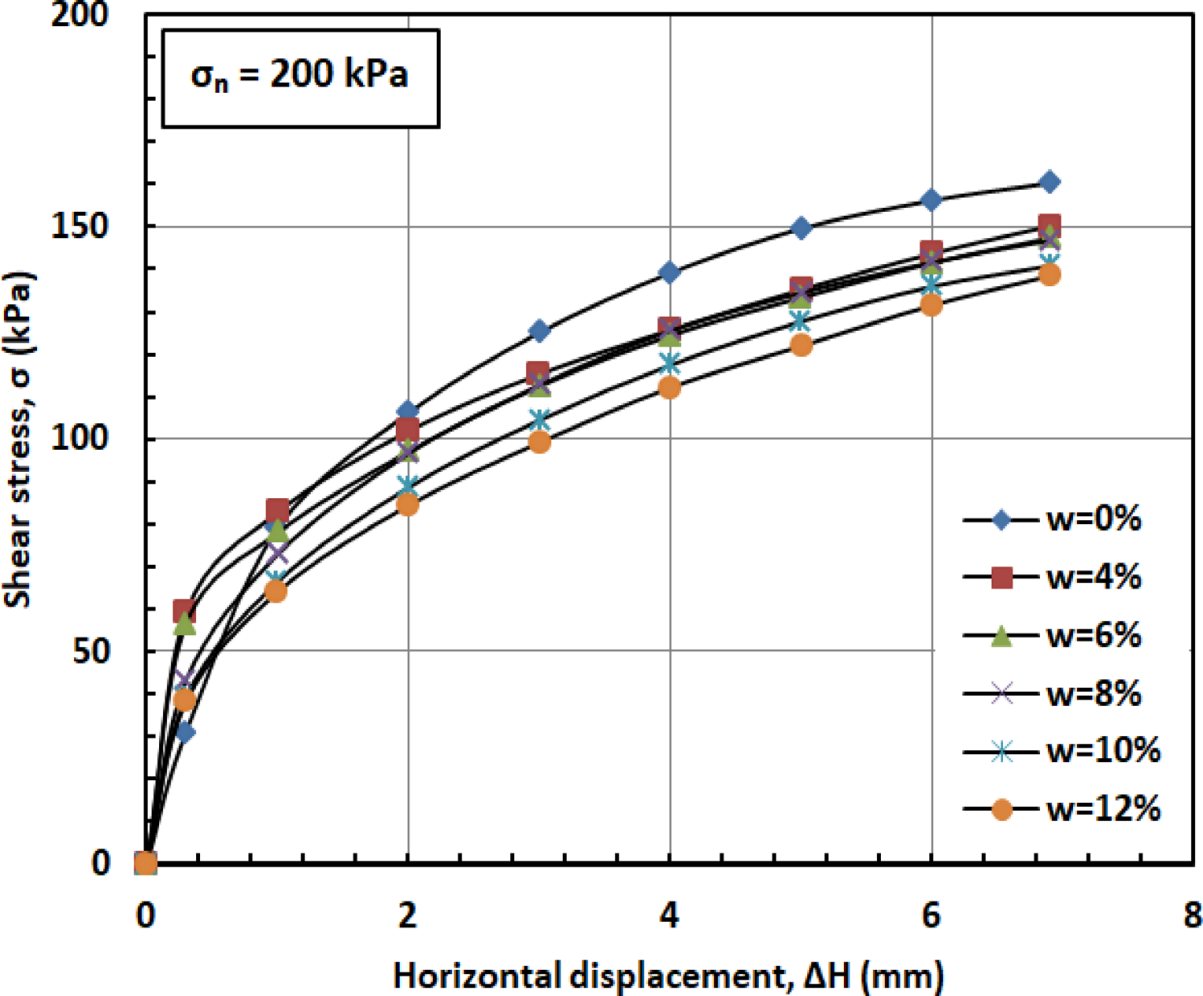
Shear strength versus horizontal displacement for 200 kPa.

**Fig. 8:**
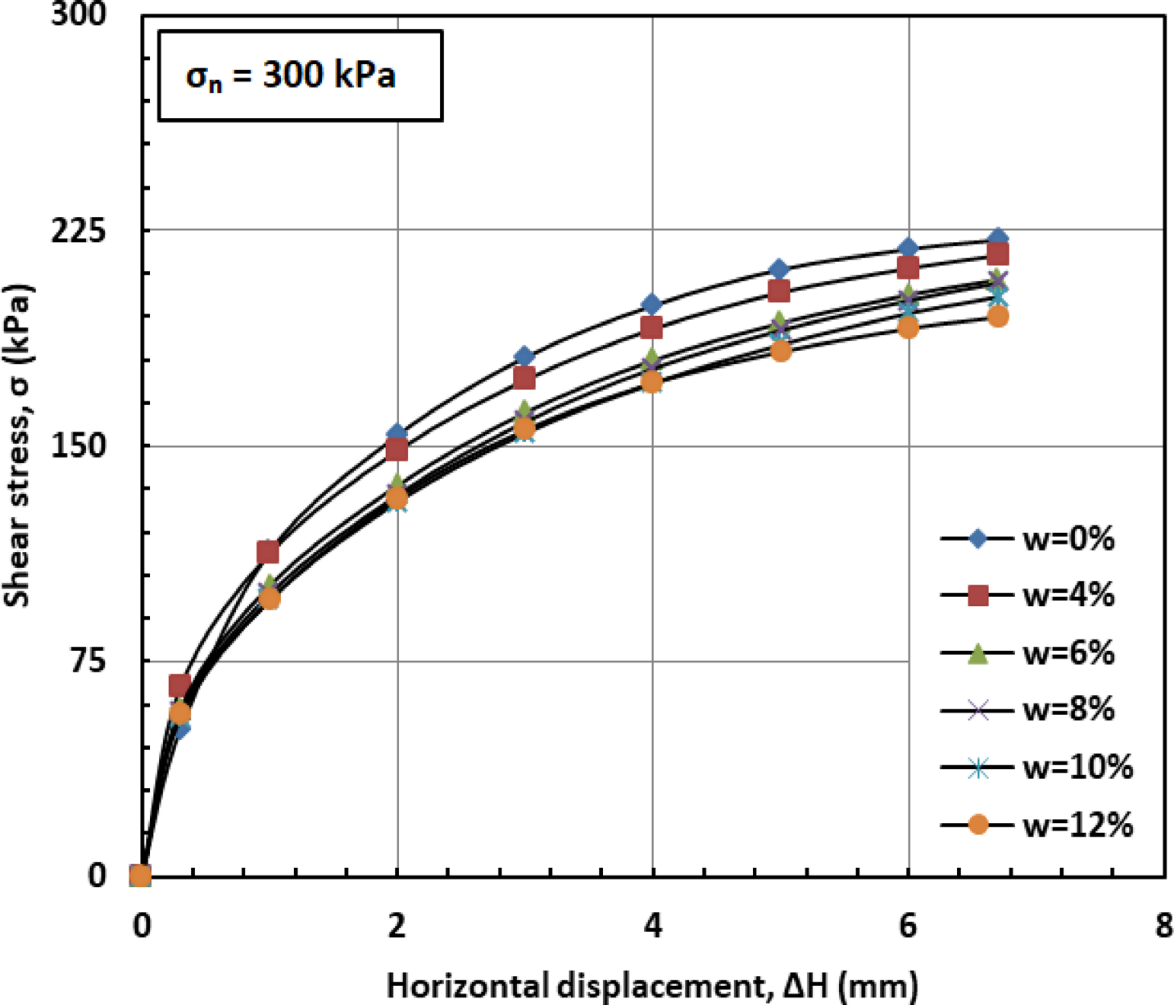
Shear strength versus horizontal displacement for 300 kPa.

Figures 6 and 7, and 8 illustrate the variation of shear stress (σ) with horizontal displacement under normal stresses (σₙ) of 100, 200, and 300 kPa. The results demonstrate a consistent decrease in shear stress (σ) as the water content (w) increases. At w = 12%, the sand–kaolin mixture shows a pronounced drop in shear stress (σ), likely due to the excessive water content significantly reducing both interparticle friction and cohesion. This results in a softer, more deformable structure where the ability of the material to resist shear stress (σ) is greatly diminished. Such behavior underscores the critical importance of controlling water content (w) in maintaining the mechanical strength of soil mixtures under load and varying normal stress (σₙ) conditions. This behavior is corroborated by recent studies. Researchers^41^ observed that increasing moisture content in silty clay leads to a decrease in shear strength due to the softening of the soil structure and reduction in interparticle friction and cohesion. Similarly, Authors in^42^ reported that higher moisture content in sand–kaolin mixtures results in decreased shear strength parameters, highlighting the importance of moisture control in soil stability. The sharp reduction in shear strength at 12% water content reflects the rapid collapse of suction and particle bonding. This trend could be quantitatively described in future work using empirical relations such as an exponential decay model of shear strength versus water content^43^, a power-law relation with degree of saturation^44^, or more recent constitutive approaches developed for unsaturated soils^45^. Such methods would provide a more mechanistic framework to complement the experimental observations.

#### Effect of normal stress

**Fig. 9:**
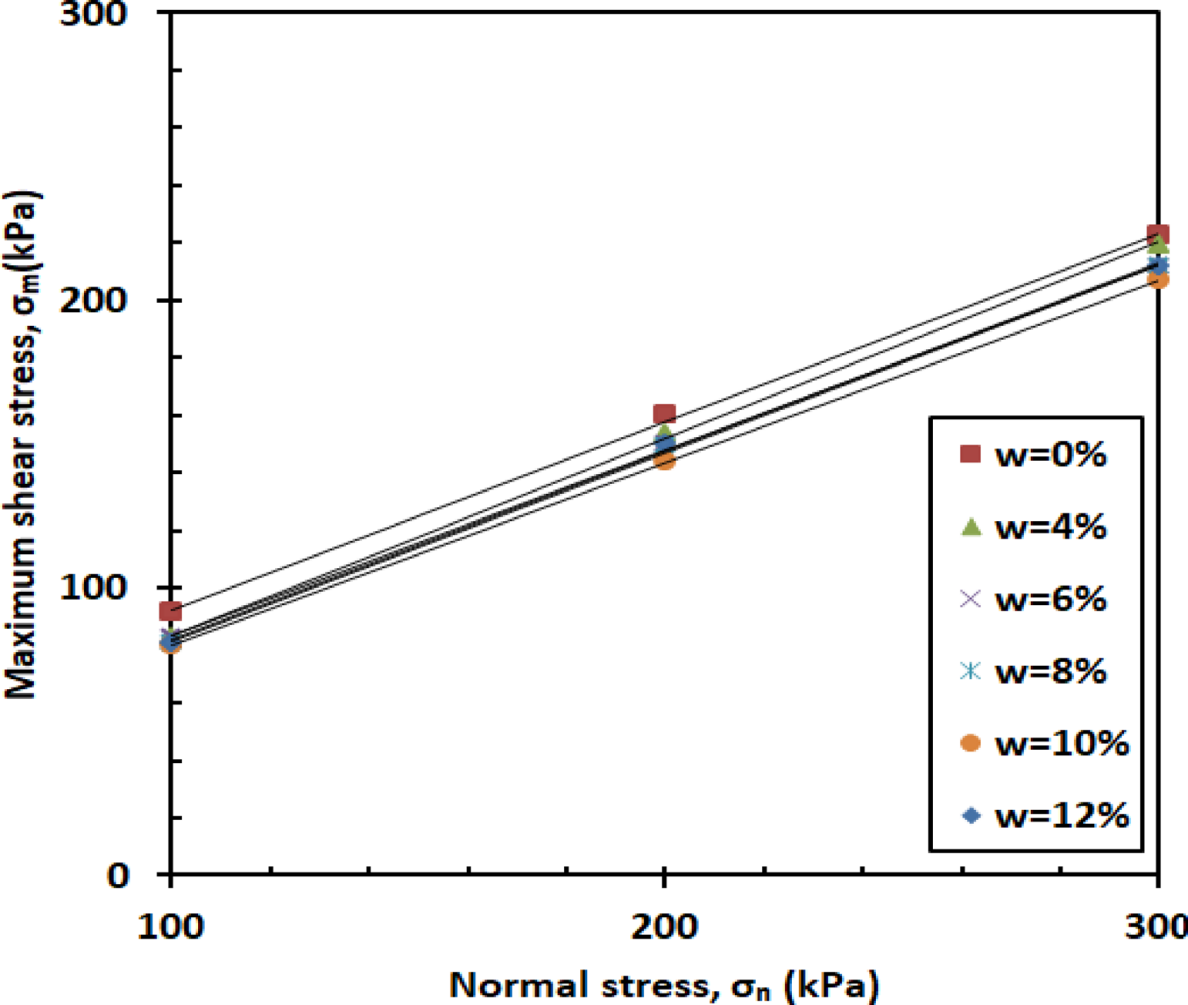
Shear strength versus normal stress.

Figure 9 shows how shear stress (σ) varies with normal stress across different levels of water content (w). As illustrated, the shear stress (σ) of the mixture increases with higher normal stress. This rise is attributed to the fact that increasing normal stress improves interparticle contact and compaction, which enhances frictional resistance and, as a result, leads to greater shear stress (σ). The rate of increase remains consistent across all moisture levels, indicating that moisture content has a limited influence on the stress–strength relationship under the tested conditions.

These enhancements in cohesion and shear strength result from the tensile resistance created by the PET strips as they act as bridging inclusions between particles to restrict relative displacement. While such a mechanism is surmised from macroscopic response, additional microstructural examination and numerical simulation are needed to discernibly observe and quantify fiber–soil interaction at the particle scale.

#### Interaction effect between normal stress and moisture content

**Fig. 10:**
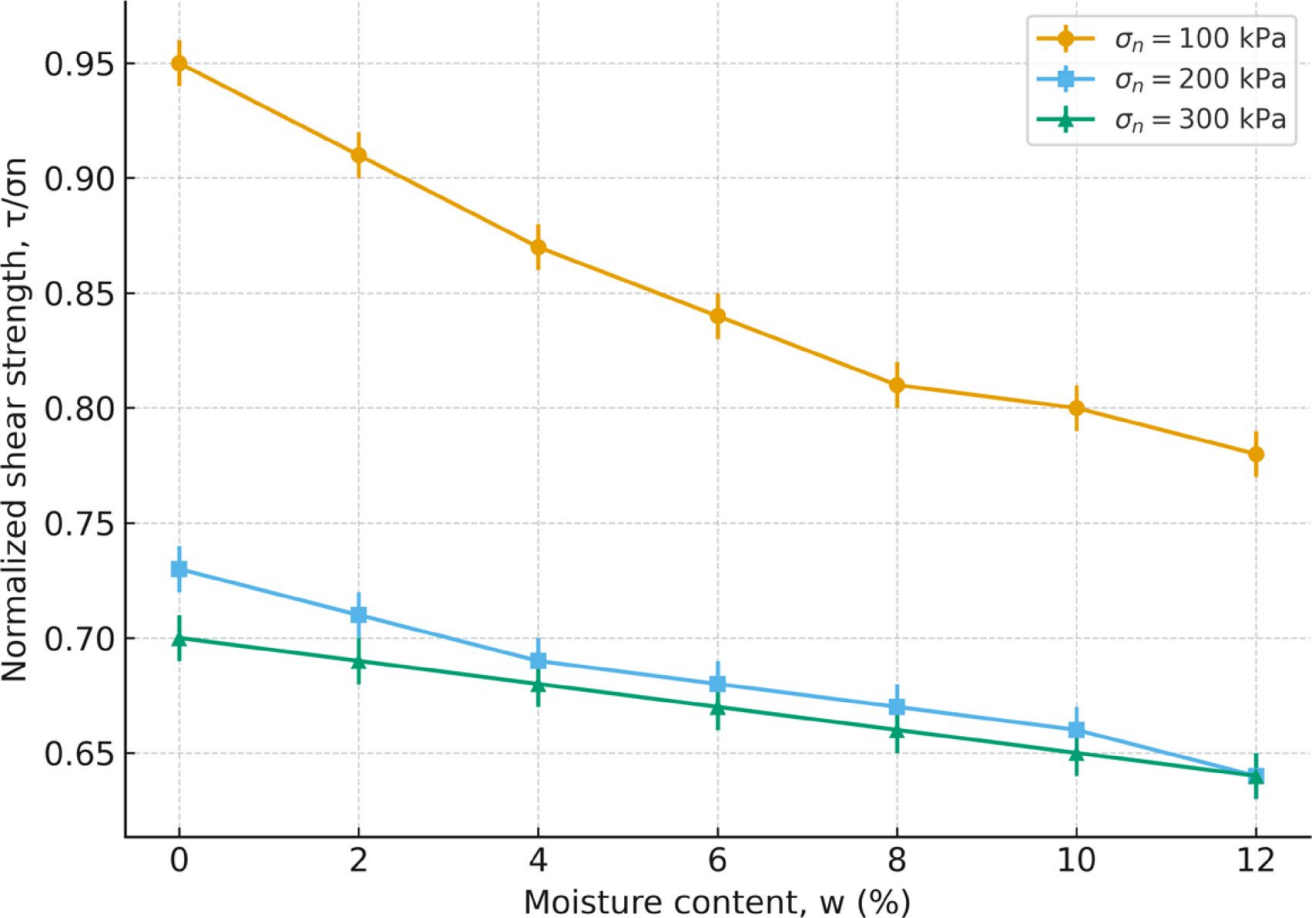
Normalized shear strength ratio (τ/σ_n_) versus moisture content (w) for different normal stresses.

To clarify the combined influence of normal stress and moisture, the normalized shear strength ratio (τ/σₙ) was plotted against water content (Fig. 10). The results show that τ/σₙ consistently decreases with increasing water content, confirming the progressive attenuation of shear resistance due to suction loss and reduced interparticle bonding. This reduction is most pronounced at lower normal stress (100 kPa), where τ/σₙ declines steeply with moisture, whereas at higher normal stresses (200–300 kPa) the reduction is less severe, indicating that greater confinement partially compensates for moisture-induced weakening. These findings quantitatively demonstrate the non-linear interplay between σ_n_ and w and highlight how excess moisture limits the capacity of the soil matrix to mobilize shear strength under applied loads.

#### Effect of moisture content on vertical displacement

**Fig. 11:**
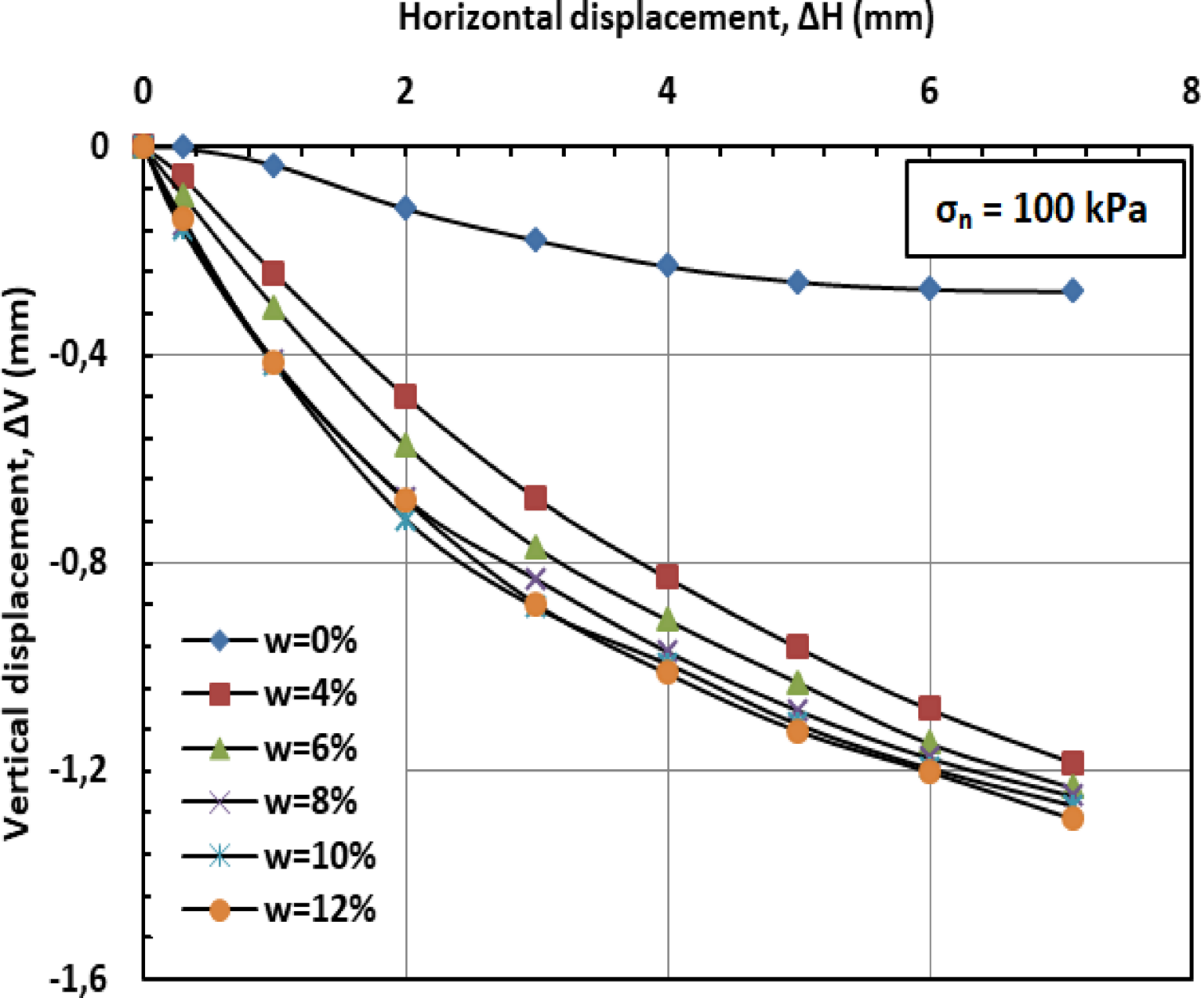
Vertical displacement versus horizontal displacement for 100 kPa.

**Fig. 12:**
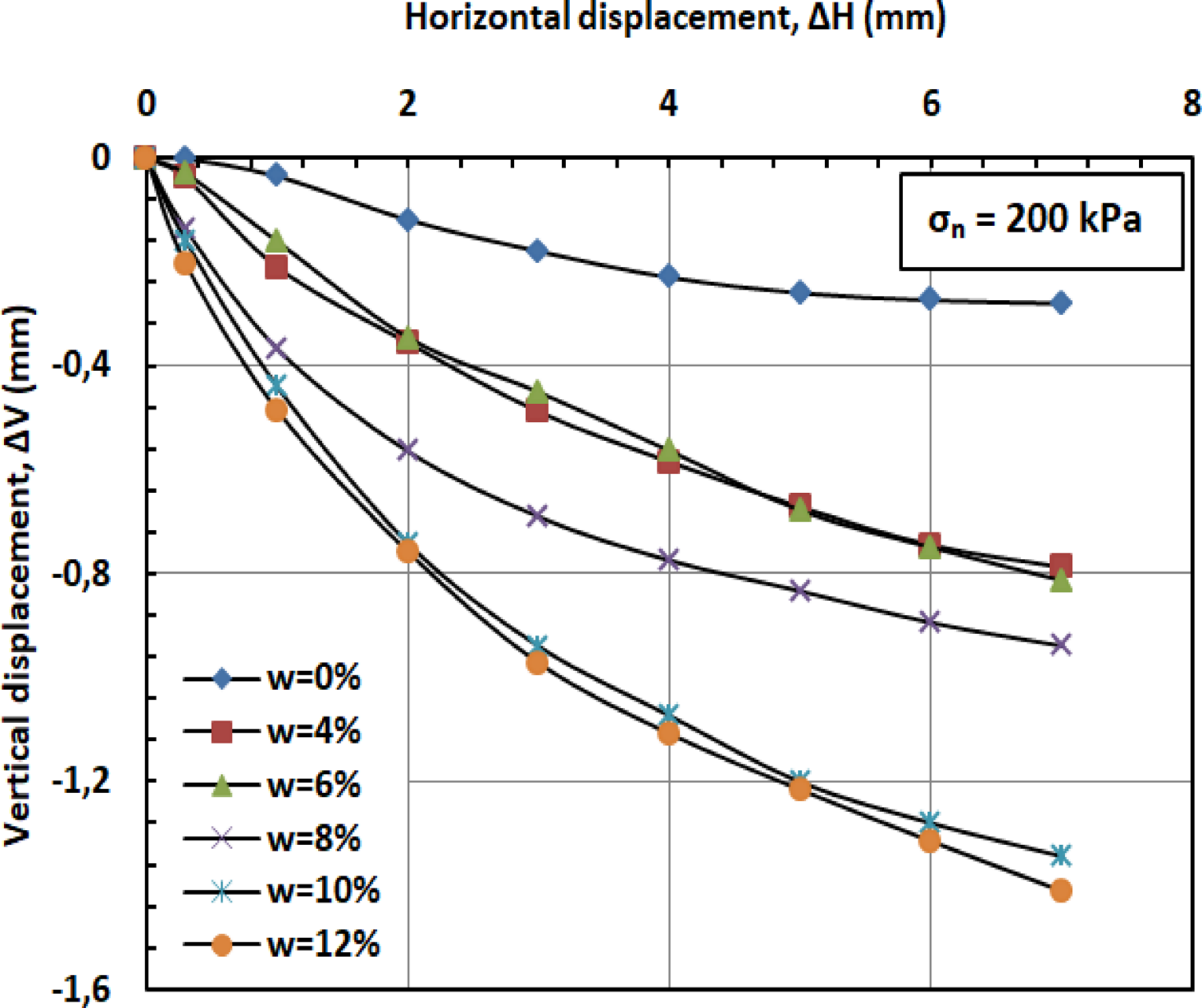
Vertical displacement versus horizontal displacement for 200 kPa.

**Fig. 13:**
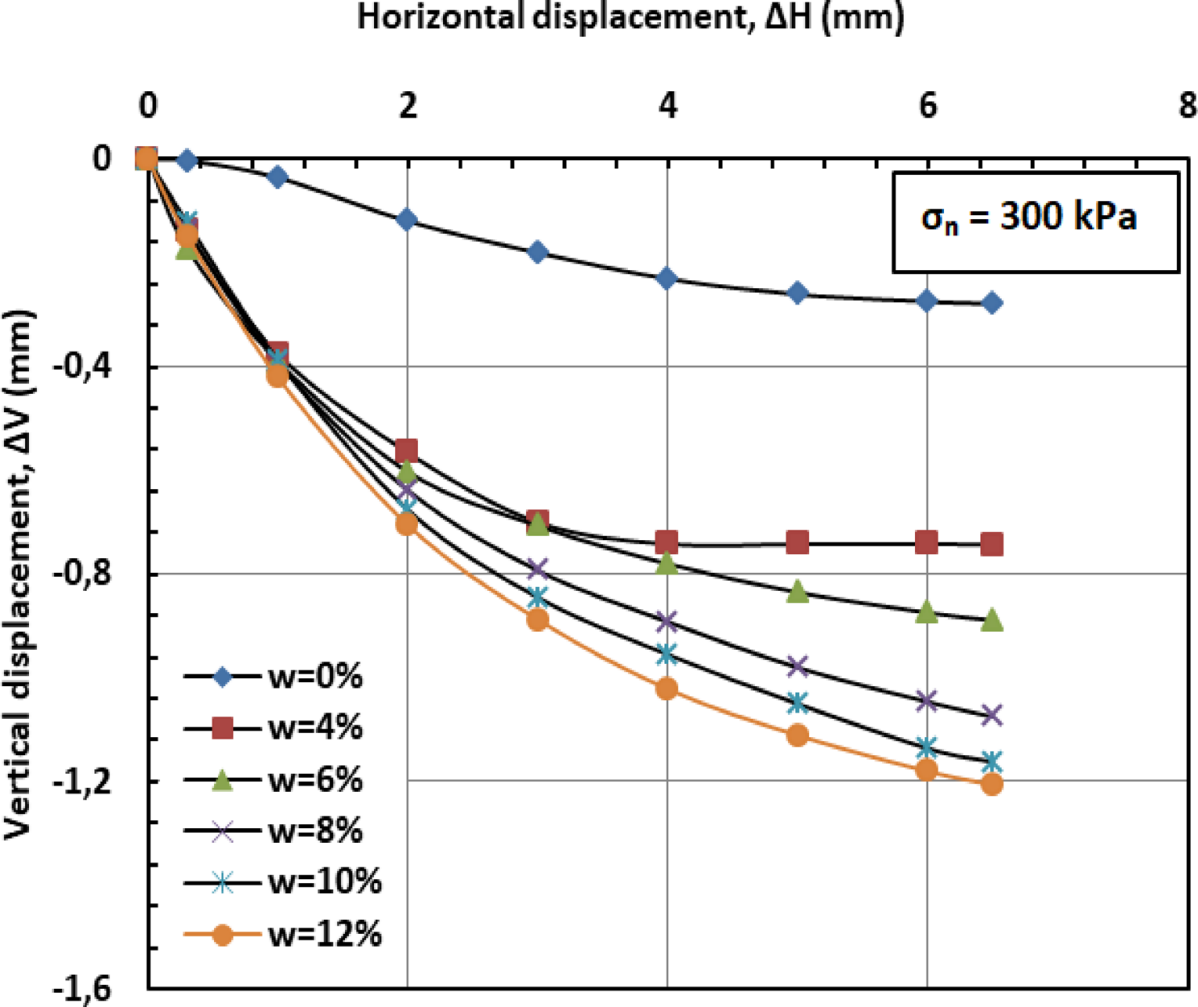
Vertical displacement versus horizontal displacement for 300 kPa.

Figures 11 and 12, and 13 present the variation of vertical displacement (Δv) with horizontal displacement (Δh) under normal stresses of 100, 200, and 300 kPa. The observed vertical displacement (ΔV) trends reflect the hybrid behavior of a sand-kaolin mixture. At 0% water content, minimal ΔV occurs due to the sand skeleton’s high frictional resistance, while the kaolin fraction contributes minor cohesion. The peak ΔV at 4–8% water content aligns with the lubrication of sand particles and kaolin’s loss of suction strength—a critical threshold where moisture softens clay bridges between sand grains^46^;^47^. Under high normal stress (300 kPa), the sand fraction dominates, suppressing ΔV through particle interlocking, as kaolin’s role diminishes under compression^48^. At very high water content (10–12%), the system behaves like a saturated sand-clay matrix, with kaolin’s plasticity mitigating further settlement^49^. These results highlight the competition between sand’s drainage capacity and kaolin’s moisture sensitivity in controlling shear-induced deformation.

#### The influence of moisture content on shear parameters

(A) Cohesion

**Fig. 14:**
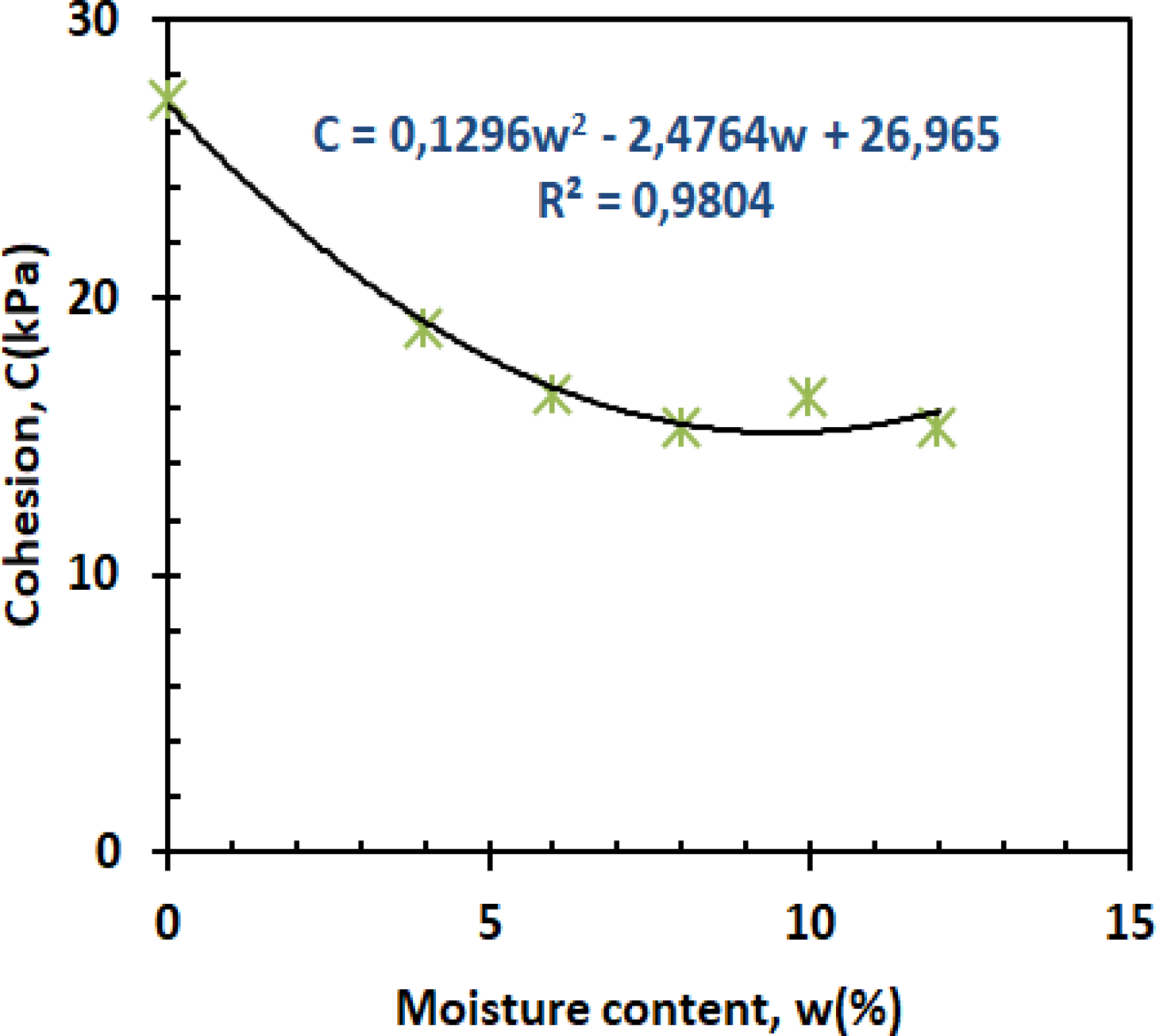
Cohesion versus moisture content.

The cohesion-moisture relationship exhibits three characteristic phases as shown in Fig. 14.

1 $$\:C=-0.1296{w}^{2}-2.4764w+26.965\:\:\:\:\:\:\:\:\:\:\:\:\:\:\:\:\:\:\:\:\:\:\:\:\:\:\:{R}^{2}=0.9804$$

The initial sharp decline (0–4% moisture) reflects capillary suction breakdown, where water disrupts kaolin’s hydrogen-bonded networks^50^. Between 4 and 10% moisture, cohesion stabilizes as residual clay bonding compensates for moisture weakening, while the slight recovery beyond 10% suggests pore water redistribution temporarily stabilizes particle contacts^51^. These trends mirror recent findings in mixed granular-clay systems, where minimum cohesion consistently occurs near the plastic limit.

(B) Friction angle

**Fig. 15:**
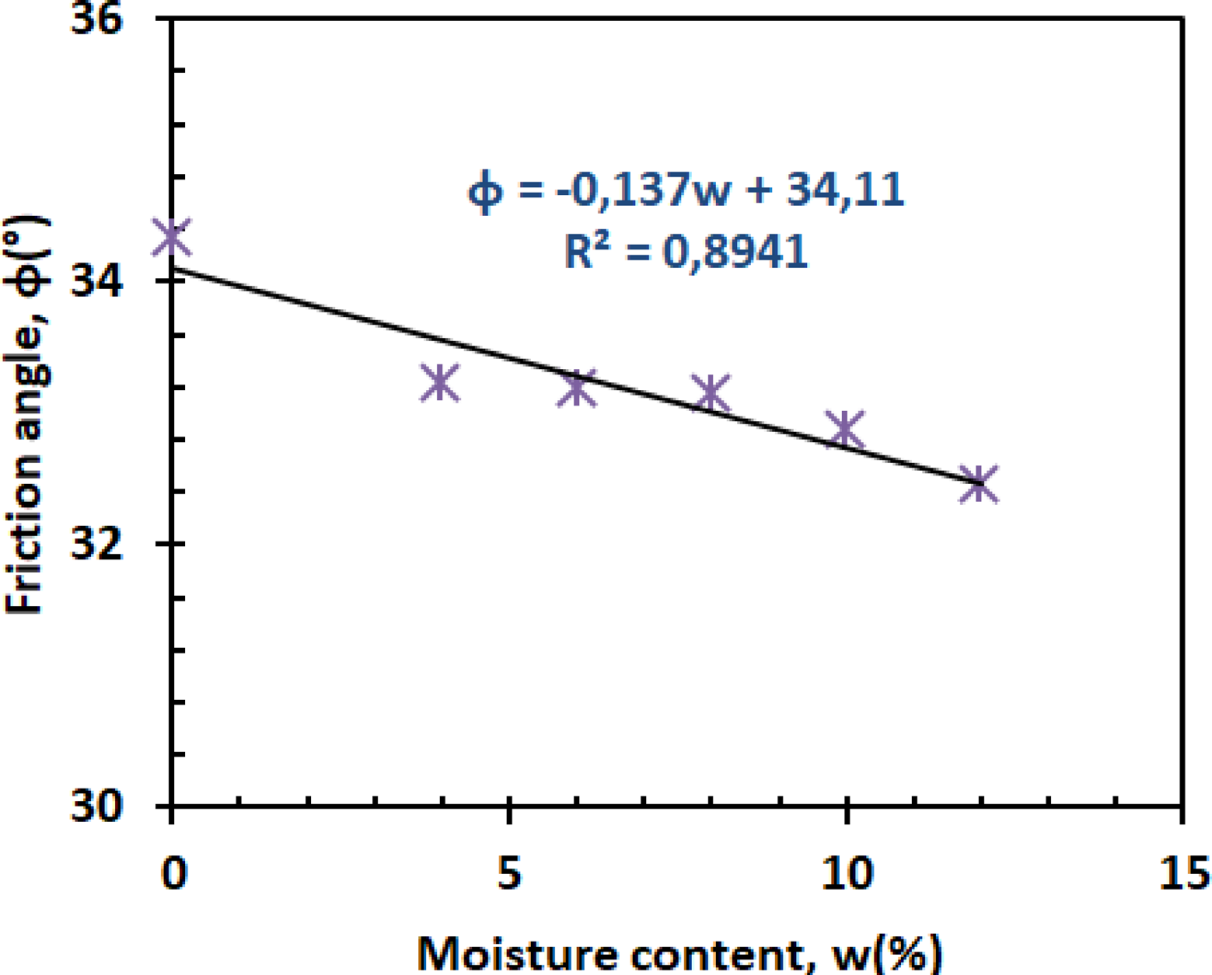
Friction angle versus moisture content.

The friction angle decreases linearly with moisture content, demonstrating water’s lubricating effect. Figure 15 presents the friction angle versus moisture content.

2 $$\:\emptyset=-0.137w+34.11\:\:\:\:\:\:\:\:\:\:\:\:\:\:\:\:\:\:\:\:\:\:\:\:\:\:\:{R}^{2}=0.8941\:$$

The preserved friction at low moisture (0–4%) results from maintained sand grain interlocking^52^, while accelerated decline beyond 8% moisture correlates with continuous water film formation at particle contacts^53^. This moisture sensitivity highlights a critical threshold where particle lubrication begins governing shear behavior - a finding consistent with contemporary studies on partially saturated soils.

### Reinforced samples

#### Effect of moisture content on shear strength

**Fig. 16:**
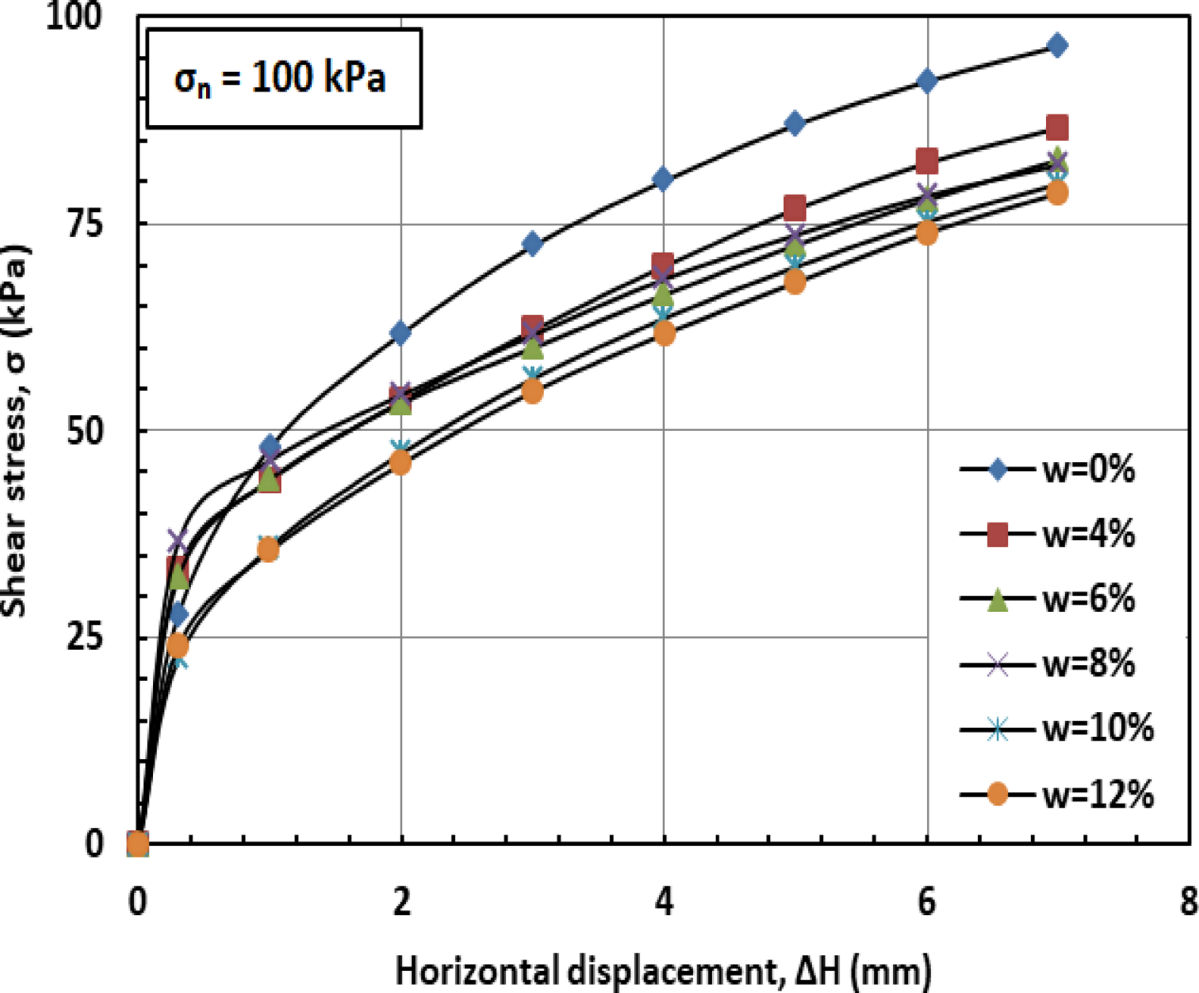
Shear strength versus horizontal displacement for 100 kPa.

**Fig. 17:**
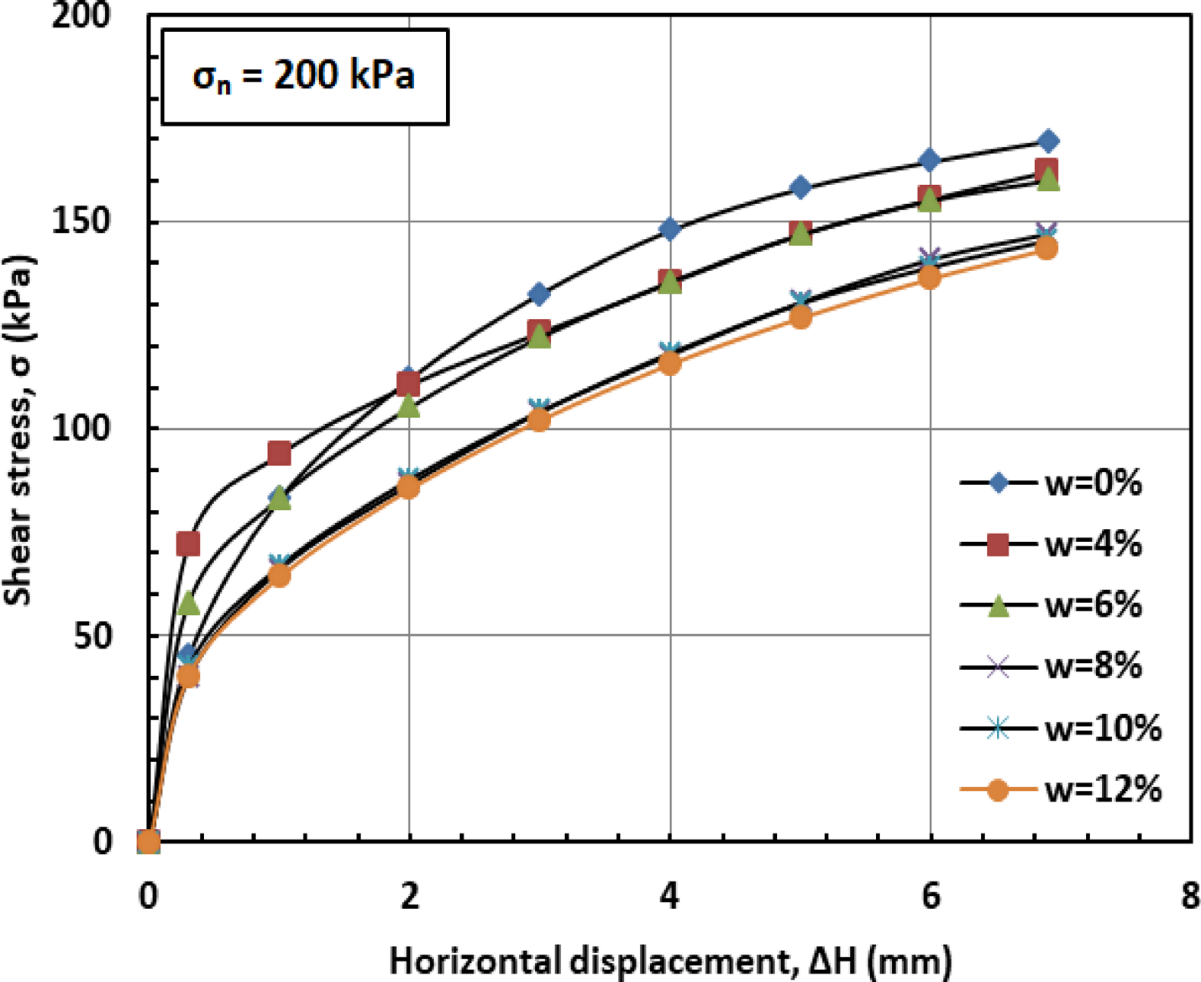
Shear strength versus horizontal displacement for 200 kPa.

**Fig. 18:**
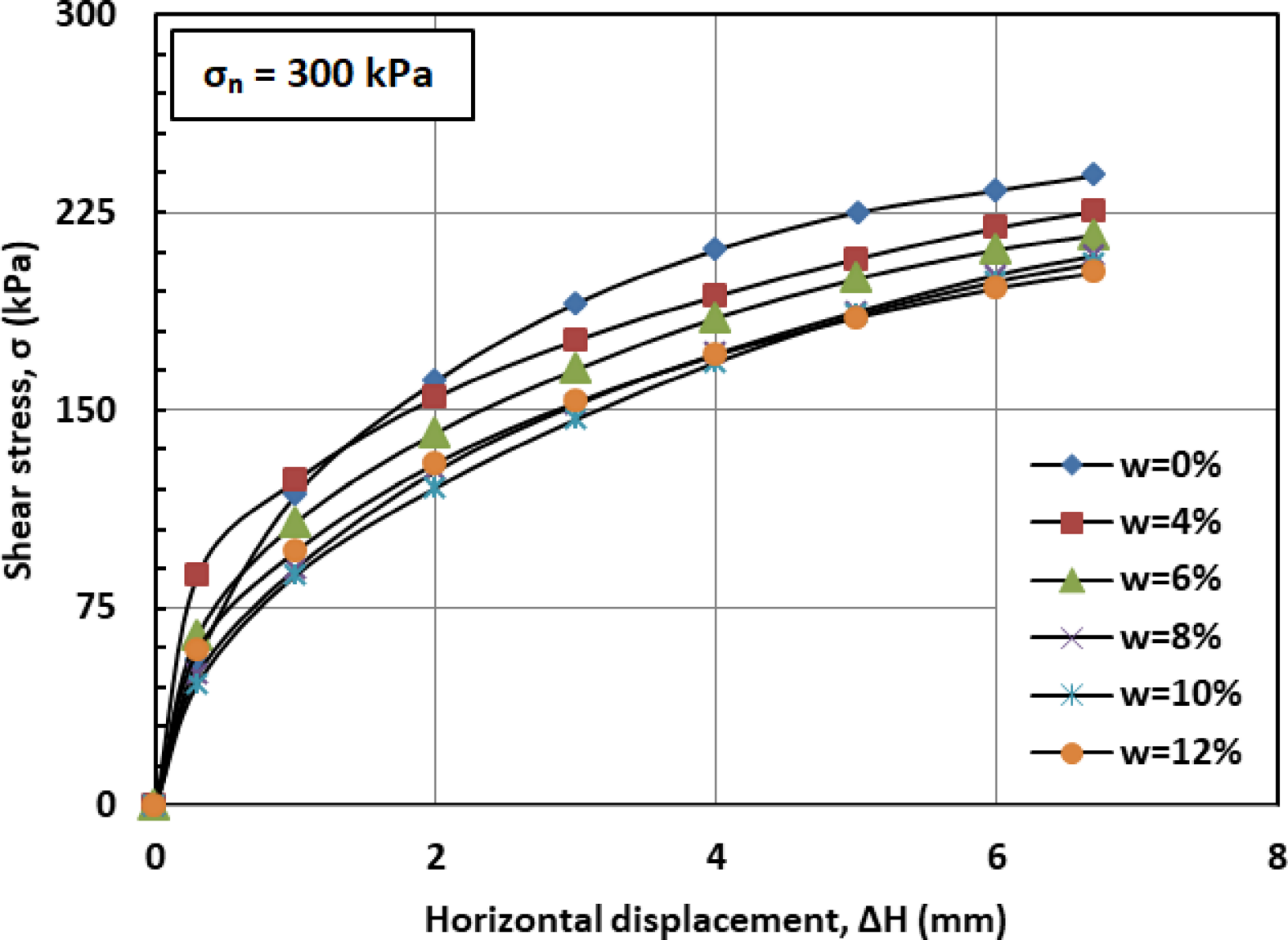
Shear strength versus horizontal displacement for 300 kPa.

Figures 16 and 17, and 18 illustrate the variation of shear stress (σ) with horizontal displacement (Δh) under normal stresses (σ_n_) of 100, 200, and 300 kPa, respectively. At 0% moisture content (w = 0%), the PET-reinforced soil mixture exhibits relatively high shear stress (σ). This resistance is primarily due to the inclusion of PET strips, which enhance cohesion (c) and overall shear resistance, while the absence of moisture preserves high friction between sand and kaolin particles. At a moisture content of 4% (w = 4%), shear stress (σ) decreases slightly as the presence of water begins to act as a lubricating agent, reducing both interparticle friction and cohesion. Despite the reduction, the PET strips continue to provide structural reinforcement, partially offsetting the moisture’s adverse effects. As the moisture content (w) increases to 6% and 8%, shear stress (σ) declines more noticeably. A phenomenon explained by the moisture-induced softening of the soil matrix, which limits the ability of the PET strips to enhance shear strength. At higher moisture levels (w = 10%–12%), shear stress (σ) typically drops further as water increasingly serves as a lubricant, significantly reducing interparticle friction and cohesion. Nevertheless, the PET strips continue to offer a degree of reinforcement, though their influence is less pronounced.

#### Effect of normal stress

**Fig. 19:**
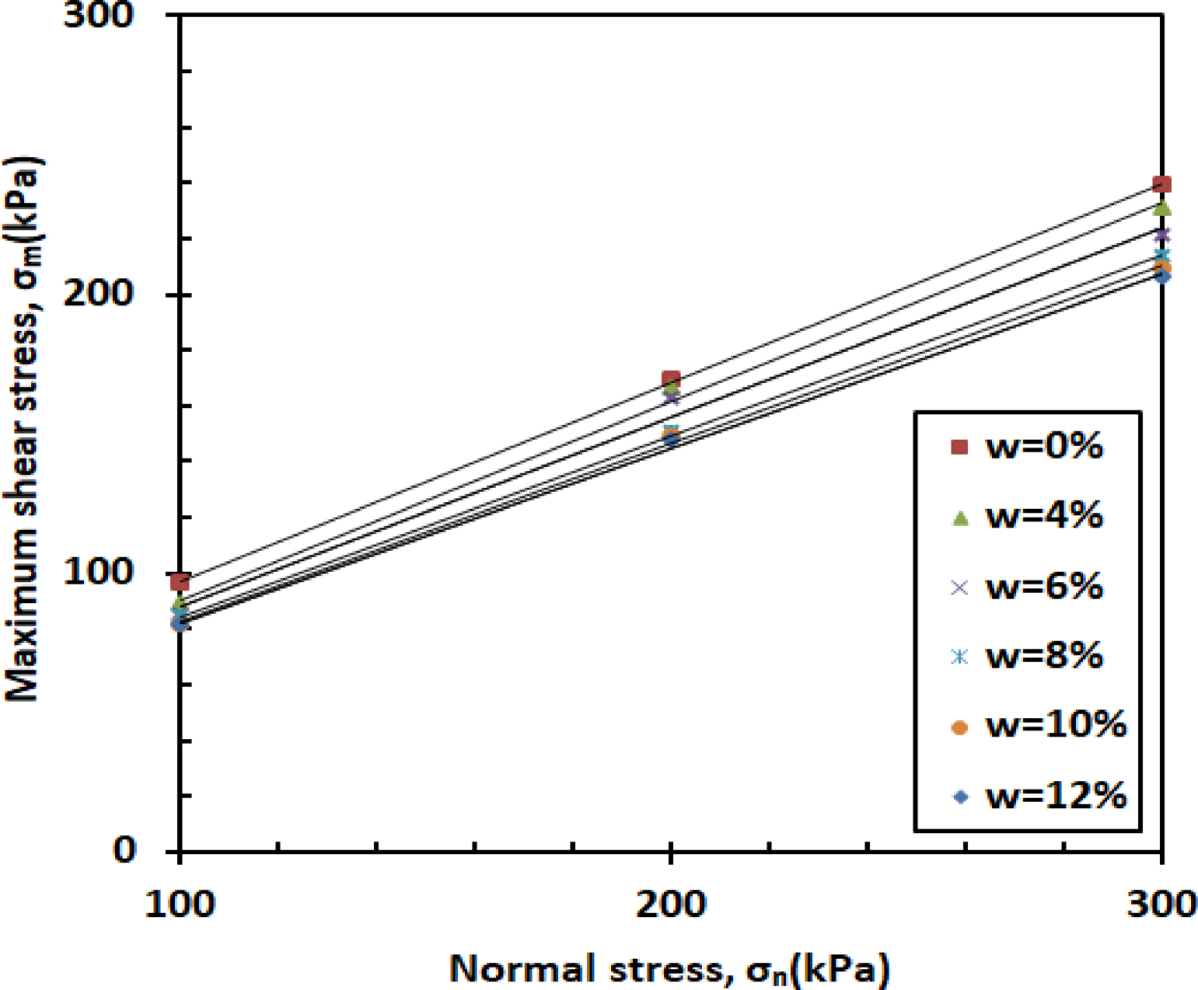
Shear strength versus normal stress.

Higher normal stresses (σ_n_) generally lead to increased interparticle contact and compaction, which contribute to greater shear stress (σ). This trend is evident across various moisture contents (w), as illustrated in Fig. 19. As the normal stress (σ_n_) increases from 100 kPa to 300 kPa, the effective stress (σ′) acting on the soil particles also rises, which enhances frictional resistance and results in higher shear stress (σ). The influence of normal stress (σ_n_) on the shear response of the sand–kaolin–PET mixture is governed by the combined effects of normal stress (σ_n_), moisture content (w), and PET reinforcement. These factors interact to define the overall mechanical behavior, strength, and stability of the reinforced soil mixture under different loading and environmental conditions.

#### Interaction between normal stress, moisture content, and PET reinforcement

The interaction between normal stress (σ_n_), moisture content (w), and PET reinforcement in the sand–kaolin–PET mixture is both complex and multifaceted. Increasing σ_n_ directly enhances shear stress (σ) by raising the effective stress (σ′) within the soil matrix; as σ_n_ rises from 100 to 300 kPa, shear strength increases proportionally in both reinforced and unreinforced specimens, reflecting greater interparticle contact and densification. Moisture content (w) exerts a decisive influence on this response by modifying cohesion (c) and friction angle (ϕ). Cohesion values decrease markedly with water addition: from ≈ 27–28 kPa at 0% moisture to 14–15 kPa at 12% moisture for unreinforced soil, while reinforced specimens retain higher values (≈ 20 kPa at 6–12% moisture), maintaining a 25–40% improvement over the unreinforced mixture. Similarly, the friction angle shows a moisture-sensitive decline. At low water contents, reinforcement provides a + 1.0–1.5° increase (e.g., 35.5° vs. 34.2° at 0% moisture), confirming the fibers’ contribution to interlocking and stress transfer. However, at higher moisture (≥ 10%), friction angles in reinforced specimens fall slightly below unreinforced mixtures (≈ 32.0° vs. 32.5°), demonstrating reduced reinforcement efficiency under strong lubrication conditions. These results illustrate the coupled influence of σ_n_ and w on reinforcement efficiency. At low to moderate w (≤ 6%), PET fibers mobilize tensile resistance effectively, slowing cohesion degradation and sustaining higher frictional resistance, which translates into greater shear strength under increasing σ_n_. By contrast, at high w (≥ 8–10%), water films weaken fiber–soil interfaces, diminishing reinforcement benefits despite the applied σ_n_. Thus, PET reinforcement is most effective in partially saturated states, where the balance between capillary bonding, interparticle friction, and fiber interlocking provides optimal resistance to shear deformation.

#### Effect of moisture content on vertical displacement

**Fig. 20:**
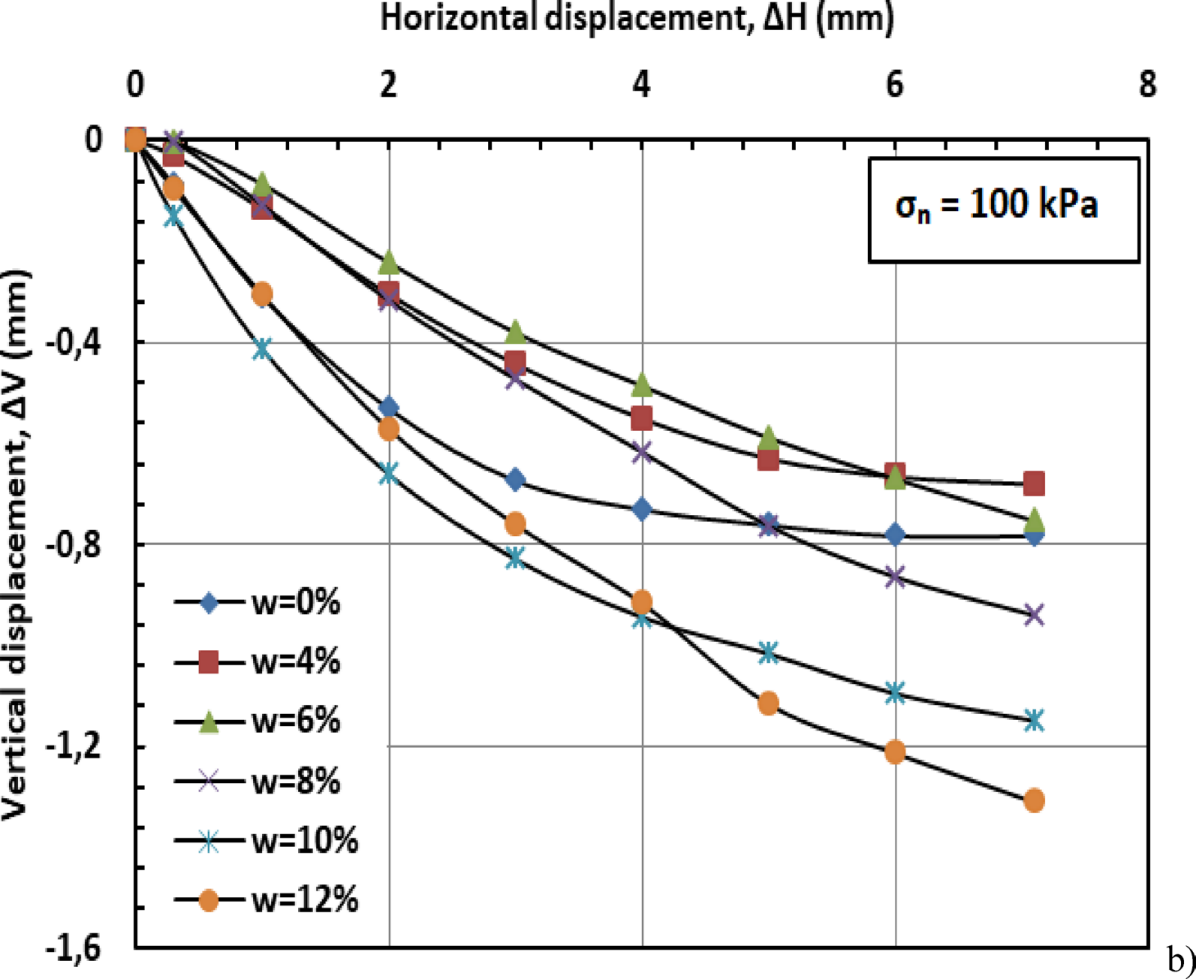
Vertical displacement versus horizontal displacement for 100 kPa.

**Fig. 21:**
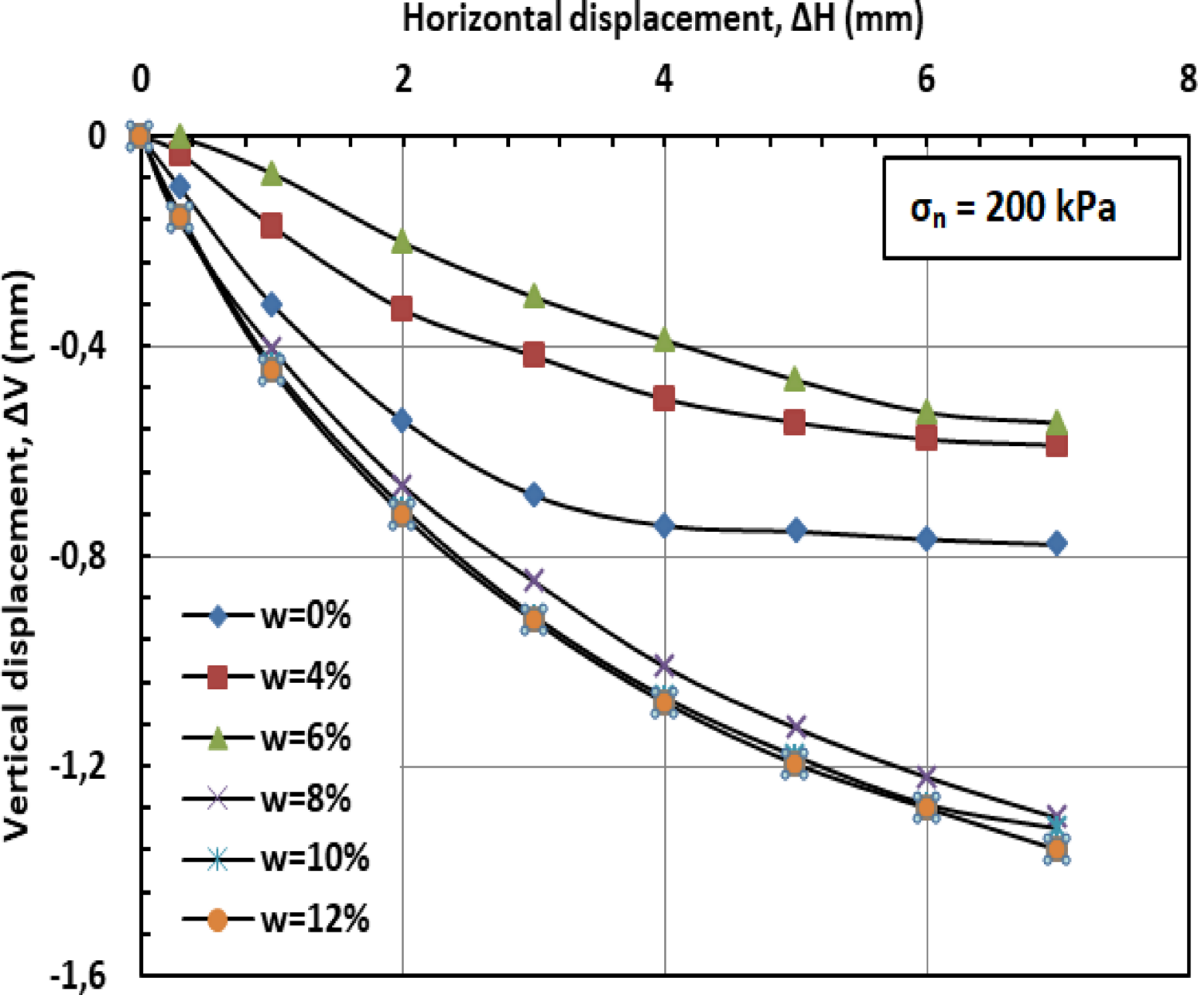
Vertical displacement versus horizontal displacement for 200 kPa.

**Fig. 22:**
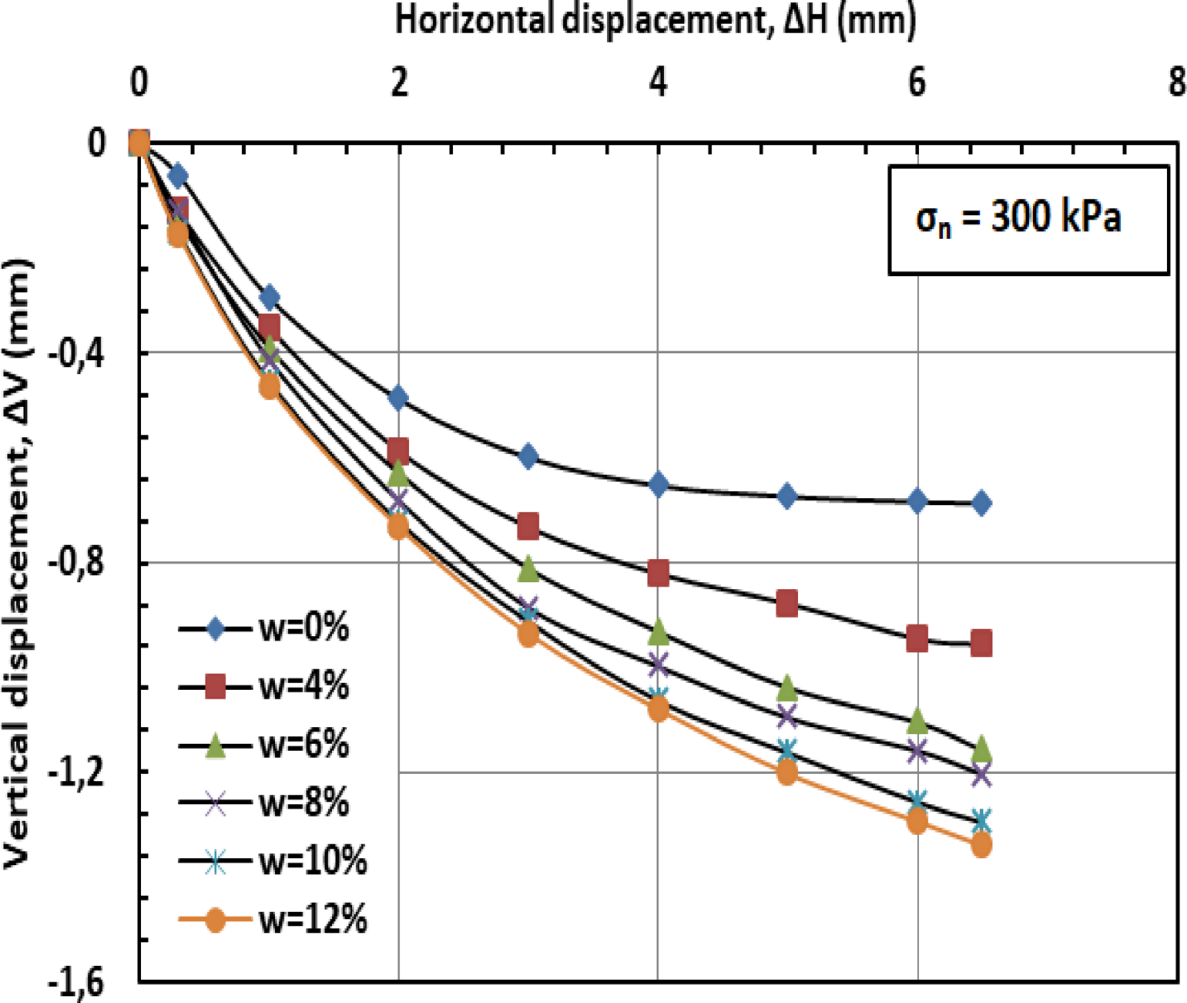
Vertical displacement versus horizontal displacement for 300kP.

Figures 20 and 21, and 22 present the variation of vertical displacement (Δv) with horizontal displacement (Δh) under normal stresses (σ_n_) of 100, 200, and 300 kilopascals (kPa), respectively. At low moisture contents (w = 0%–6%), the limited presence of water results in minimal lubrication, leading to increased interparticle friction and higher resistance to shear deformation. Consequently, the mixture displays lower vertical displacement (Δv). At medium to high moisture contents (w = 8%–12%), the presence of water molecules reduces friction between particles, facilitating their movement and resulting in greater vertical displacement (Δv). When the moisture content (w) becomes excessive, the soil may approach conditions of liquefaction, particularly under high stress or deformation. This leads to a significant reduction in effective stress (σ′) and results in increased vertical displacement (Δv).

#### Effect of PET reinforcement

PET strips act as reinforcement elements within the sand–kaolin mixture, significantly enhancing its overall mechanical stability and resistance to shear deformation. These strips create a network-like structure within the matrix, which helps distribute applied loads more uniformly and effectively resist vertical displacement (Δv). The inclusion of PET reinforcement mitigates the influence of moisture content (w) on vertical displacement by providing additional internal cohesion and structural support. Even at elevated moisture levels (w = 10%–12%), the PET strips maintain the integrity of the soil structure, limiting excessive displacement that typically results from reduced interparticle friction and effective stress (σ′). Furthermore, PET reinforcement contributes to preserving the stiffness of the soil–polymer mixture, thereby reducing deformation under shear loading. This increased stiffness plays a crucial role in enhancing the load-bearing capacity of the reinforced soil and in preventing significant vertical settlements, especially in conditions where water-induced softening would otherwise compromise soil performance.

#### Synergistic effect

The combined influence of PET reinforcement and low moisture content (w) plays a crucial role in minimizing vertical displacement (Δv) in the sand–kaolin mixture. The PET strips provide mechanical reinforcement by resisting deformation and enhancing the internal cohesion of the soil matrix. Simultaneously, a reduced moisture content diminishes the lubricating effect of water, thereby limiting interparticle slippage and maintaining higher interparticle friction. This synergy results in improved mixture stability and a notable reduction in vertical displacement, even across a range of moisture conditions. As the moisture level increases, the PET reinforcement becomes increasingly important in offsetting the potential loss in shear resistance and stiffness typically induced by water infiltration.

#### Influence of moisture content on shear parameters

(A) Cohesion

**Fig. 23:**
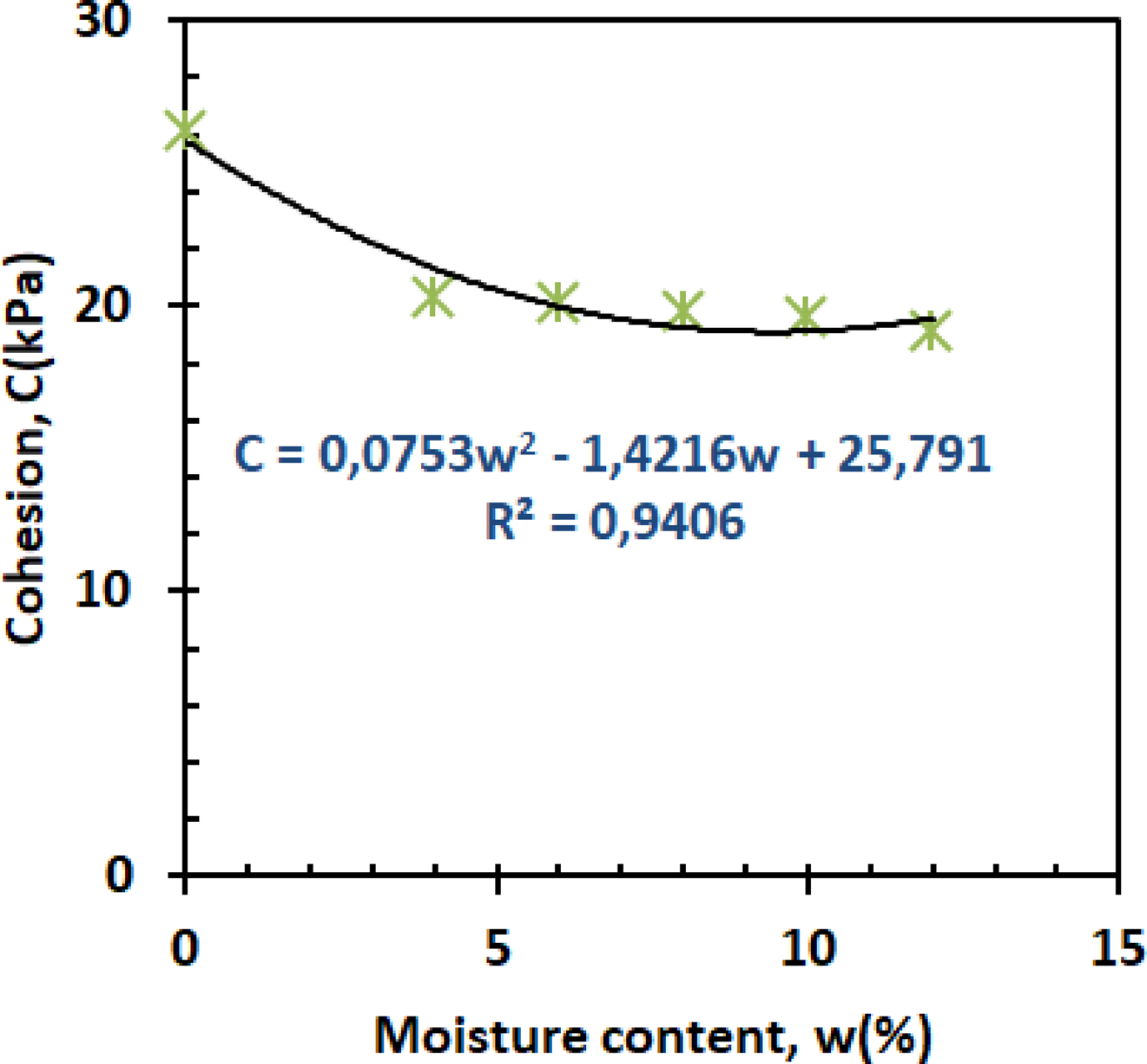
Cohesion versus moisture content.

Figure 23 illustrates the variation in cohesion (c) as a function of moisture content (w).

3 $$\:C=0.0753{w}^{2}-1.4216w+25.791\:\:\:\:\:\:\:\:\:\:\:\:\:\:\:\:\:\:\:\:\:\:\:\:\:\:\:{R}^{2}=0.9406$$

At a dry state (w = 0%), the reinforcing effect of PET strips is particularly significant. The PET strips interlock effectively with sand and kaolin particles, enhancing cohesion by providing structural support and facilitating particle bonding. In this condition, PET reinforcement greatly increases cohesion by reducing the relative movement between particles, as there is no moisture to lubricate the soil particles. At moderate moisture levels (w = 4%–6%), the lubricating effect of water slightly reduces the effectiveness of PET reinforcement, leading to a reduction in cohesion (c). However, the PET strips still contribute positively to cohesion, as they provide internal support and enhance particle bonding, especially at moisture levels where a balance between lubrication and cohesion exists. At higher moisture contents (w = 8%–12%), the lubricating effect of water becomes more pronounced, further reducing cohesion. Despite this, PET reinforcement continues to improve cohesion by promoting bonding between soil particles and PET fibers, although the reinforcement’s effectiveness is less than under dry conditions.

(B) Friction angle

**Fig. 24:**
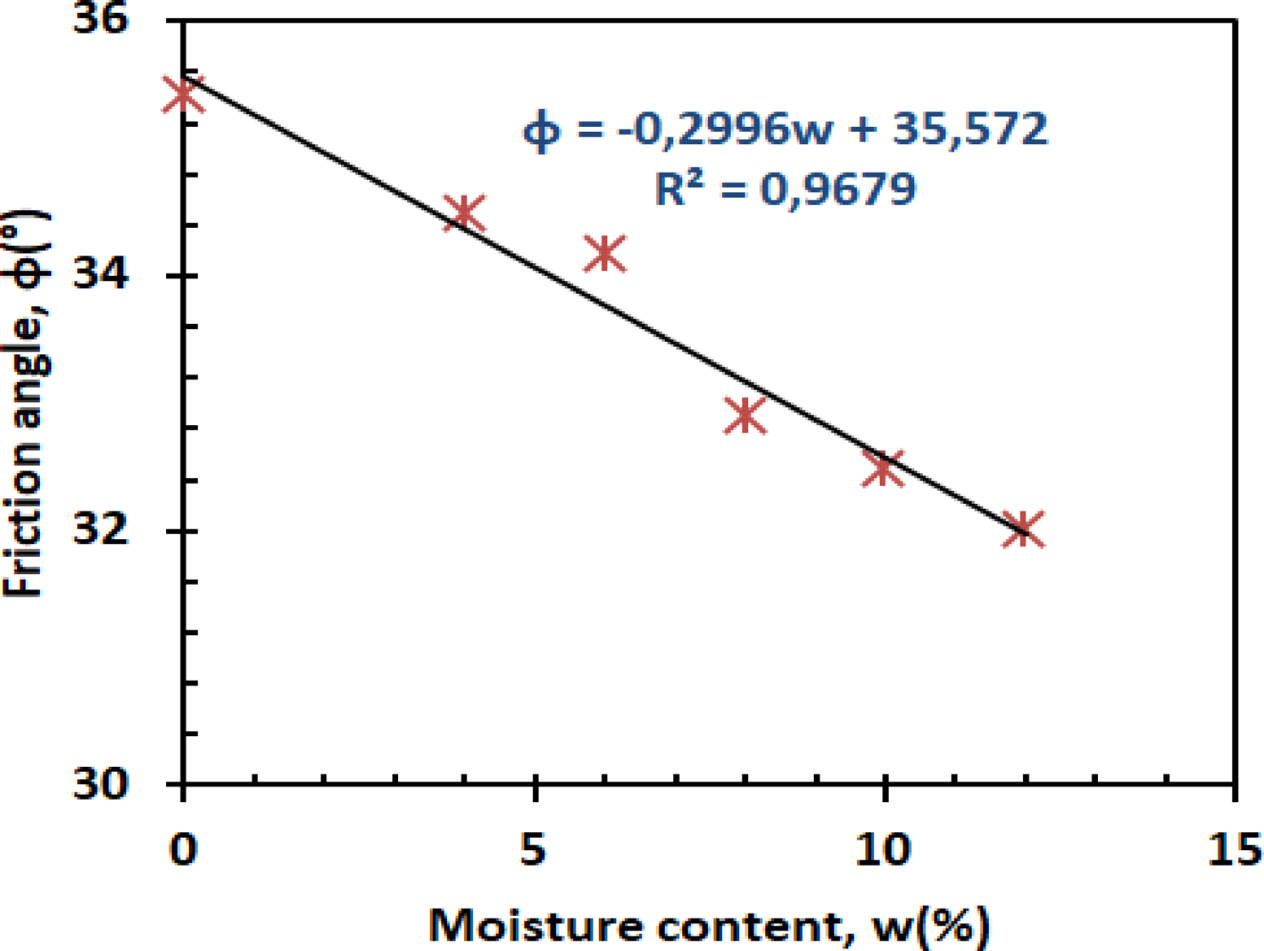
Friction angle versus moisture content.

Figure 24 depicts the linear variation of the friction angle (φ) with moisture content (w).

4 $$\:\emptyset=-0.2996w+35.572\:\:\:\:\:\:\:\:\:\:\:\:\:\:\:\:\:\:\:\:\:\:\:\:\:\:\:{R}^{2}=0.9679$$

At low moisture levels (w = 0%–4%), the friction angle of the reinforced mixture remains relatively stable. The PET strips contribute structural support and promote interlocking between the sand and kaolin particles, which helps to maintain a consistent friction angle by increasing interparticle friction. As the moisture content increases to moderate and high levels (w = 6%–12%), the lubricating effect of water reduces interparticle friction, which can lead to a decrease in the friction angle (φ). However, the presence of PET reinforcement partially counteracts this reduction by enhancing the bonding between soil particles and PET strips, providing additional resistance to shear deformation and helping to maintain the friction angle to a certain extent.

### Comparative evaluation

#### Maximum shear stress

**Fig. 25:**
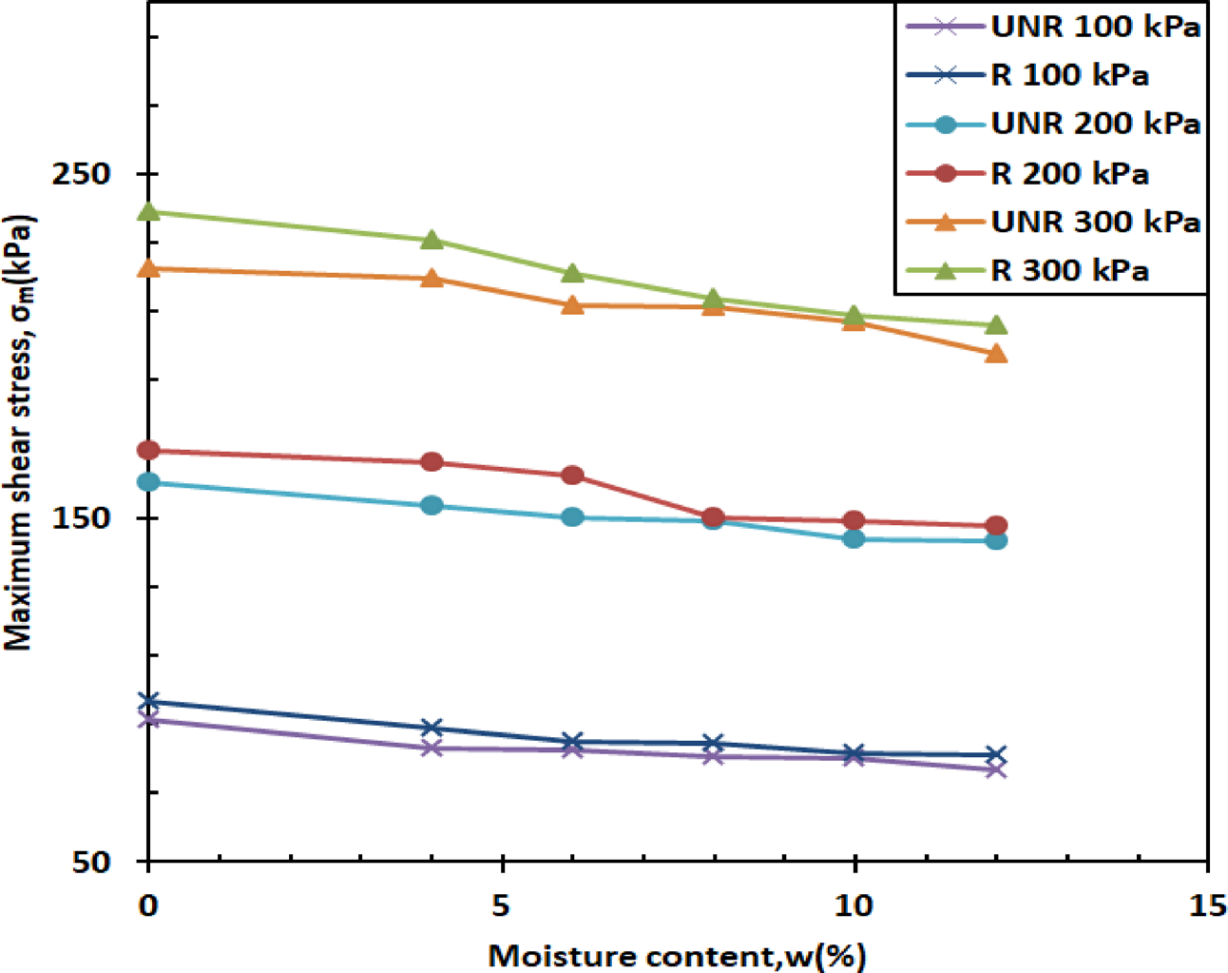
Maximum shear strength us a function of moisture content.

The experimental results demonstrate that PET strip reinforcement significantly enhances the shear strength of sand-kaolin mixtures across all tested moisture contents (0–12%) and normal stresses (100–300 kPa). For unreinforced specimens, the maximum shear stress (τ_max_) decreases progressively with increasing moisture content, exhibiting a 45–50% reduction at w = 12% (e.g., from 145 kPa to 90 kPa under σ_n_ = 300 kPa). This behavior aligns with recent findings in^54^, who identified capillary bridge dissolution and particle lubrication as primary weakening mechanisms in sand-clay systems. In contrast, PET-reinforced specimens maintain consistently higher shear strength, retaining 20–25% greater τ_max_ values compared to unreinforced samples under identical conditions. The reinforcement efficacy stems from two key mechanisms: tensile resistance provided by PET strips, which remains effective regardless of moisture content^55^, and enhanced particle confinement that limits moisture-induced particle separation^56^. The reinforcement benefit ratio (RBR = τ_max_,_rein_f/τ_max_, _unrein_f) reveals stress-dependent performance, decreasing from 1.40 at σ_n_ = 100 kPa to 1.22 at σ_n_ = 300 kPa, indicating greater relative improvement under lower stresses where PET’s tensile contribution dominates^57^. These findings underscore PET reinforcement’s practical value for geotechnical applications exposed to moisture fluctuations, particularly in slope stabilization (optimal RBR = 1.30–1.40 at w = 8–12%) and subgrade reinforcement (τ_max_ retention > 65% under high stresses). The results provide quantitative evidence that PET strips effectively counteract moisture-driven strength loss while improving overall shear performance in sand-kaolin composites. Figure 25 presents maximum shear strength as a function of moisture content.

#### Shear parameters

**Fig. 26:**
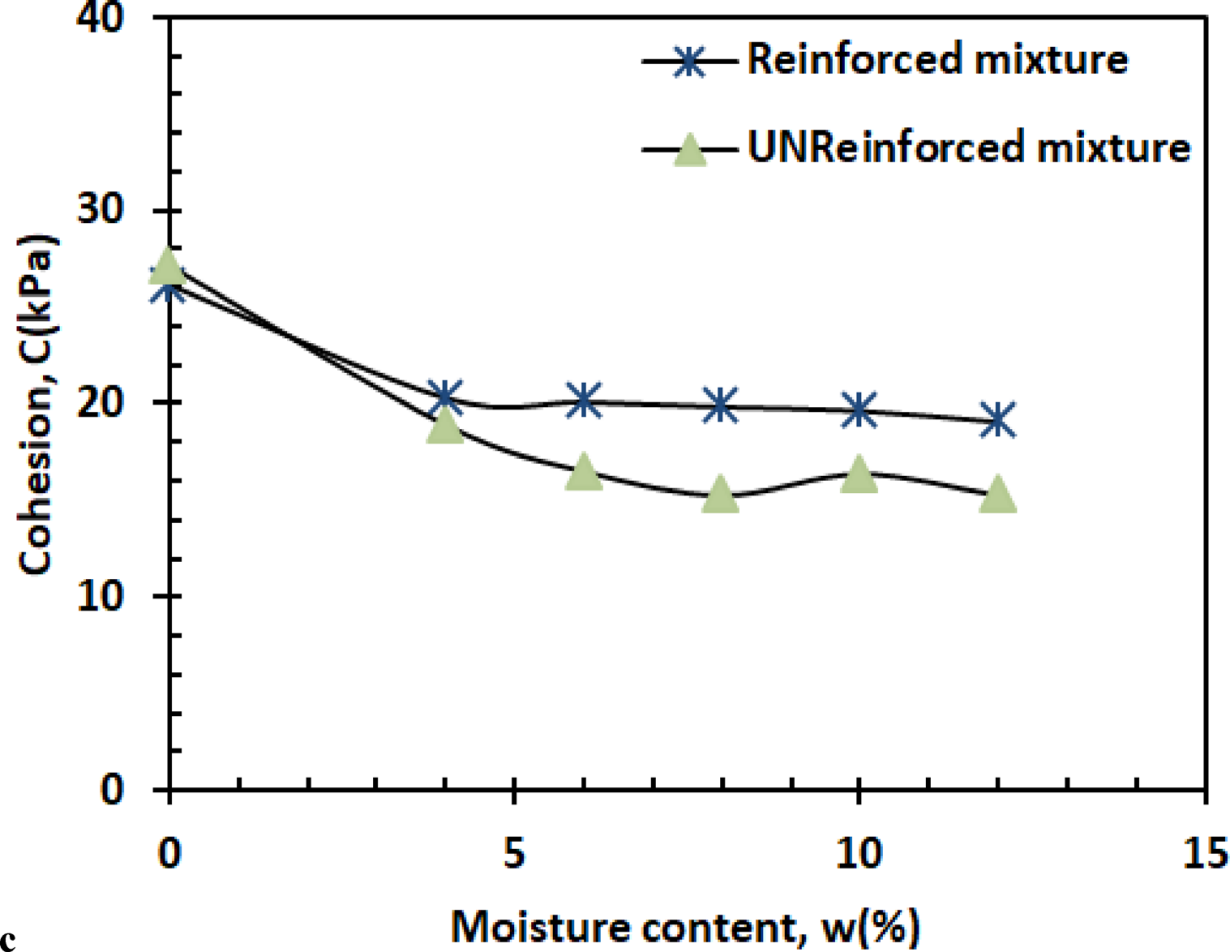
Variation of friction angle as a function of moisture content for both reinforced and unreinforced sand–kaolin mixtures.

**Fig. 27:**
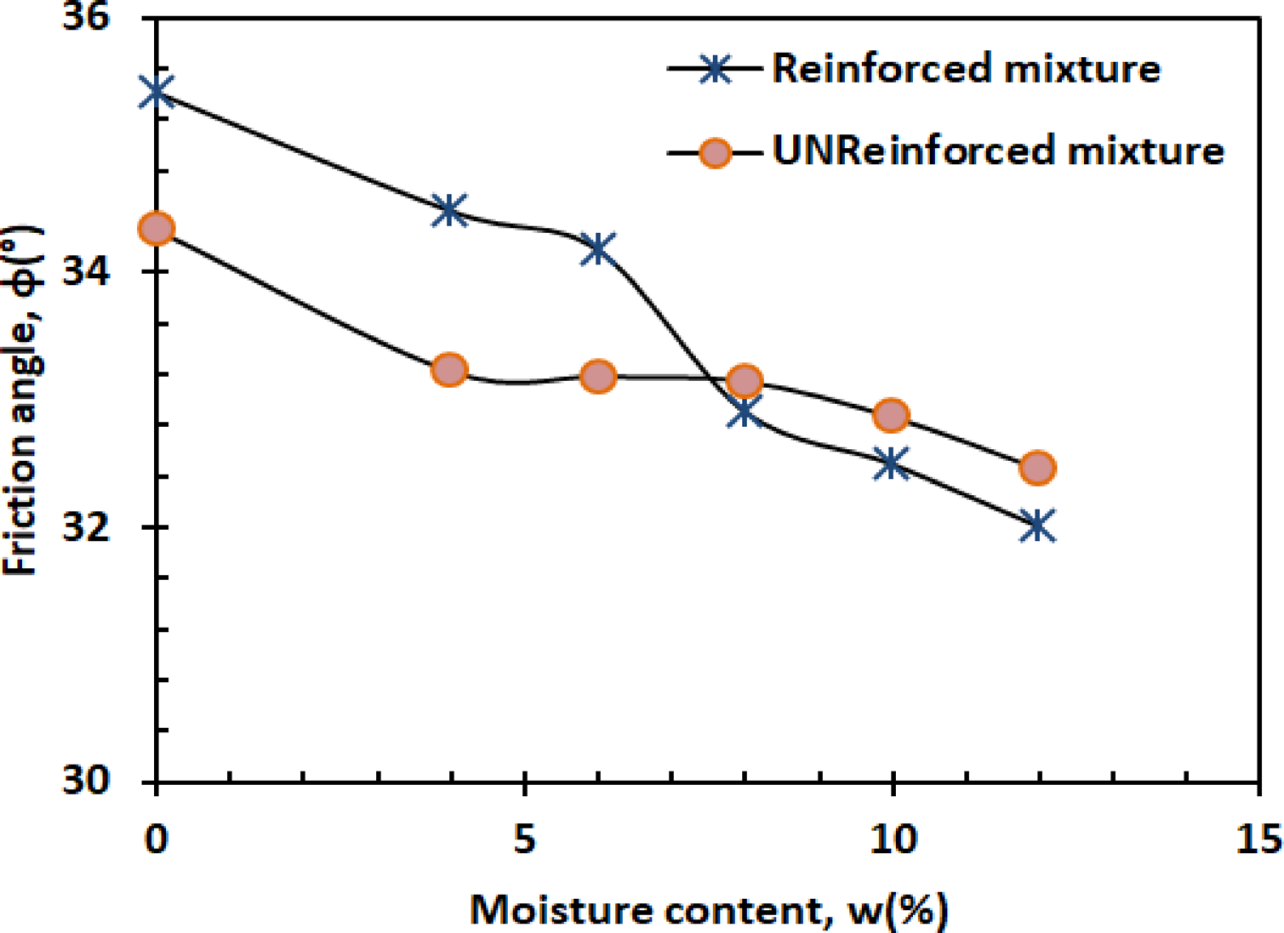
Variation of cohesion and friction angle, respectively, as a function of moisture content for both reinforced and unreinforced sand–kaolin mixtures.

Figures 26 and 27 present the variation of cohesion and friction angle, respectively, as a function of moisture content for both reinforced and unreinforced sand–kaolin mixtures. These figures allow a direct comparison of how PET strip reinforcement influences the shear strength parameters under varying water contents.

#### Reinforced vs. unreinforced cohesion behavior

The cohesion enhancement from PET reinforcement remains significant across the entire moisture range (Fig. 26), with reinforced specimens maintaining 25–40% higher values than unreinforced soil. At 0% moisture, cohesion is ≈ 27 kPa (reinforced) vs. 28 kPa (unreinforced), showing a comparable initial state. At 4% moisture, reinforced samples reach ≈ 20 kPa compared to ≈ 18 kPa for unreinforced, corresponding to a 12% increase. The maximum cohesion difference occurs at 6% moisture (≈ 20 kPa vs. ≈13 kPa, i.e., a 7 kPa gap and a 35–40% improvement), demonstrating PET’s effectiveness during critical moisture transitions. Even at higher moisture levels (10–12%), reinforced mixtures stabilize around 19–20 kPa while unreinforced mixtures drop to 14–15 kPa, sustaining a 25–30% higher cohesion. This performance stems from the fibers’ ability to maintain tensile forces between soil particles even as water weakens natural bonds, a phenomenon recently quantified through digital image correlation studies^58^. While both mixtures eventually lose cohesion at high moisture, the reinforced soil’s slower decline rate suggests PET fibers help preserve some particle connectivity despite lubrication effects.

#### Friction angle dynamics

The friction angle behavior reveals a moisture-dependent reinforcement mechanism (Fig. 27). At 0% moisture, reinforced specimens exhibit ≈ 35.5° compared to ≈ 34.2° for unreinforced soil, a + 1.3° increase (≈ 4% improvement). Below 6% moisture, PET fibers increase ϕ by 1.0–1.5° through enhanced particle interlocking, as demonstrated in discrete element modeling of fiber-reinforced sands^59^. However, above 8% moisture, the advantage diminishes: at ≈ 8% moisture, both mixtures converge at ≈ 33.0°, and at higher contents (10–12% moisture, unreinforced mixtures maintain ≈ 32.5° vs. ≈32.0° for reinforced, a 0.5° higher value). This transition indicates that excessive water compromises fiber–soil stress transfer. The effect correlates with recent findings on interface shear behavior, where water films exceeding 2 μm thickness reduce fiber–soil friction coefficients by 15–20%^60^. The results emphasize PET’s optimal operational window for friction enhancement in moderately moist conditions (w ≤ 6%).

## Conclusion

This study investigated the shear behavior of sand–kaolin mixtures composed of 65% sand and 35% kaolin, reinforced with 0.6% PET strips, under varying moisture contents ranging from 0 to 12%. Its primary contribution lies in providing a quantitative assessment of the combined effects of moisture variation and PET strip reinforcement on the mechanical response of fine-grained sand–clay mixtures. Based on the experimental results, the following conclusions highlight the role of moisture content in governing the shear behavior of both unreinforced and PET-reinforced sand–kaolin mixtures.

- Increasing moisture content (0–12%) led to a progressive reduction in shear strength and friction angle, while vertical displacement increased due to lubrication effects.
- PET reinforcement (0.6% strips) consistently improved shear performance, with the strongest benefits observed under dry and moderately moist conditions (w ≤ 6%).
- At σ_n_ = 100 kPa and w = 0%, reinforced mixtures reached 60 kPa shear strength versus 50 kPa for unreinforced samples; at w = 12%, reinforcement still provided an advantage (35 vs. 25 kPa), though with reduced efficiency.
- Cohesion values were consistently higher in reinforced mixtures across all moisture levels, confirming the stabilizing effect of PET strips.
- The friction angle response was moisture-dependent: PET reinforcement enhanced φ at low w, but unreinforced mixtures exhibited slightly higher φ at high w (8–12%) due to reduced soil–fiber interfacial contact.
- PET strips are thus effective in improving the shear strength and cohesion of sand–kaolin mixtures under controlled moisture conditions, but their frictional efficiency diminishes in saturated states.
- These findings highlight the need to consider moisture sensitivity when designing reinforced soil.

Future works include scaling up this work to pilot-scale laboratory tests and possible field applications of PET-reinforced soils, and determining long-term durability under cyclic wetting–drying and other environmental loading conditions. More research can investigate the impact of different PET geometries, aspect ratios, and higher reinforcement contents for maximum performance. Furthermore, integration of experimental evidence with numerical modeling and microstructural analysis would provide further understanding of fiber–soil interaction mechanisms and contribute to the development of rational design practices for sustainable soil reinforcement.

## Data Availability

The datasets used and/or analyzed during the current study are available from the corresponding author on reasonable request.

## Abbreviations

PET: Polyethylene terephthalate
OMC: Optimum moisture content
RMC: Residual moisture content
SWCC: Soil-water characteristic curve
USCS: Unified soil classification system
